# Ancient Egyptian Mummified Bodies: Cross-Disciplinary Analysis of Their Smell

**DOI:** 10.1021/jacs.4c15769

**Published:** 2025-02-13

**Authors:** Emma Paolin, Cecilia Bembibre, Fabiana Di Gianvincenzo, Julio Cesar Torres-Elguera, Randa Deraz, Ida Kraševec, Ahmed Abdellah, Asmaa Ahmed, Irena Kralj Cigić, Abdelrazek Elnaggar, Ali Abdelhalim, Tomasz Sawoszczuk, Matija Strlič

**Affiliations:** a Heritage Science Laboratory Ljubljana, Faculty of Chemistry and Chemical Technology, University of Ljubljana, Večna pot 113, Ljubljana 1000, Slovenia; b Institute for Sustainable Heritage, University College London, 14 Upper Woburn Place, London WC1H 0NN, U.K.; c Department of Microbiology, Institute of Quality Sciences and Product Management, Krakow University of Economics, Henryka Sienkiewicza 4, Krakow 30-033, Poland; d The Egyptian Museum in Cairo, Cairo 4272083, Egypt; e Faculty of Archaeology, Ain Shams University, Abbasiya Cairo 11566, Egypt

## Abstract

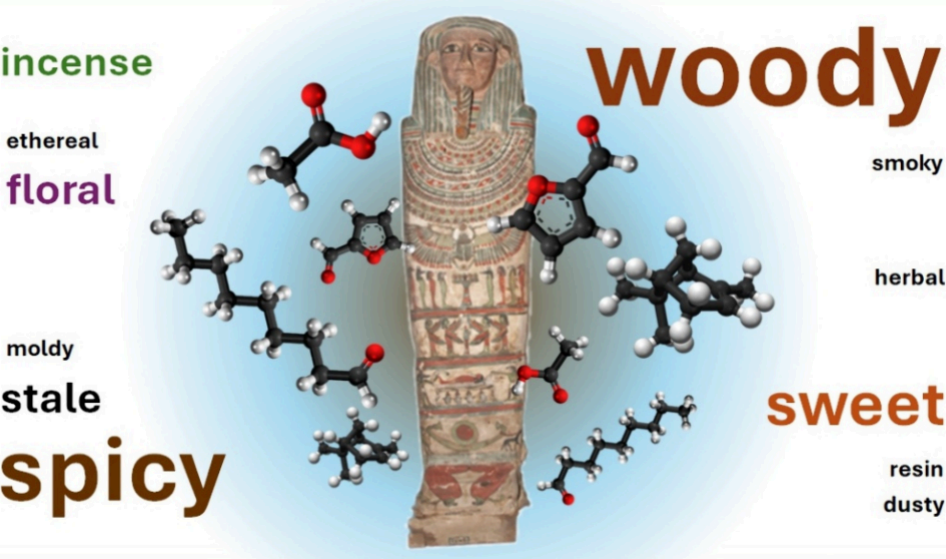

Ancient Egyptian mummification was a mortuary practice aimed at preserving the body and soul for the afterlife, achieved through a detailed ritual of embalming using oils, waxes, and balms. While most research on Egyptian mummified bodies has so far been conducted in European collections, our study focuses on the collection of the Egyptian Museum in Cairo. The goal was to evaluate whether contemporary smells reflect the mummification materials and, if so, what information can be of value to collection interpretation and conservation. We combined panel-based sensory analyses with gas chromatography-mass spectrometry-olfactometry (GC-MS-O), microbiological analysis, and historical and conservation research. Apart from differences in odor intensity, the sensory analyses highlighted common olfactory descriptors for all samples: “woody”, “spicy”, and “sweet”. GC-MS-O identified four categories of volatiles based on their origin: (i) original mummification materials; (ii) plant oils used for conservation; (iii) synthetic pesticides; and (iv) microbiological deterioration products. However, the use of insect repellents similar in composition to the original mummification materials makes it challenging to attribute the origin of some compounds. Clusters based on the chemical and olfactory profiles of the smells emerged, suggesting similarities based on the archeological period, conservation treatments, and materiality.

## Introduction

The sense of smell is fundamental to our daily lives, but its role in archeology and heritage has thus far been marginal. The history of the past is often presented to the public as odorless, despite the value of smell for artifact interpretation.^1,2^ Smells from heritage objects also hold a scientific value, as they can be used to obtain information about the original material, degradation pathways and rates, as well as conservation and restoration treatments and to better understand and interpret the heritage significance.

Ancient Egyptian mummification materials and techniques are a topic of continued interest, as demonstrated through recent publications.^3−6^ Furthermore, the smell of mummified bodies has historically attracted a lot of attention from experts and the general public, with sensory descriptions ranging from fragrant to foul. The disparity in perceptual experiences has been interpreted as evidence of the “mummy’s symbolism of both immortality and death”.^7^

While academic studies explored embalming through residue analysis, no study in non-European collections has thus far focused on a collection and analysis of volatile emissions from mummification materials and mummified bodies and their relation with the perceived smell. Conservators have reported the smell of mummified bodies as pleasant, possibly due to balms and resins, but this has never been systematically investigated.

Mummified bodies provide invaluable insights into ancient Egyptian civilization, offering unique opportunities to explore aspects of health, disease, environment, and religious practices.^8^ Mummification was not only a social practice and practical method of preserving the body from putrefaction but also a ritual with spiritual significance closely interwoven with religious beliefs.^9^ The preservation of the body was crucial to the successful transition of the soul into the afterlife. Here, smells are indicators of the state of purity or corruption of the body. A “good” (intended as pleasant) smell was associated with the bodies of deities, in contrast to dead bodies. Various ancient funerary and medical texts use terms to express the importance of mummification in view of the processes of decay, such as 
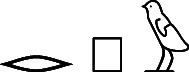
 (rpw: rot), 
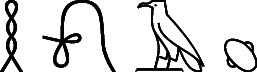
 (hw3t: putrefaction), and 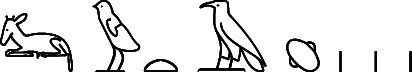
(iwtyw: corruption).^10^

Over time, mummification practices varied greatly, initially reserved for royalty, then gradually extended to the lower socio-economic classes, and eventually becoming accessible to the majority of the population.^11^ Social hierarchy was reflected in the quality of burial practices, with the pharaohs and elite members receiving the most expensive and high-quality mummification. In addition, the quality of mummification techniques also differed between and within the historical periods and within workshops using slightly different recipes and balms according to the age, gender, and body part.^12,13^ A detailed description of the mummification process is reported in the Supporting Information, SI1.

During natural mummification in Predynastic Egypt (c. 5000 BCE), the body was preserved by exposure to the hot, dry desert sand. The Old Kingdom (c. 2700–2200 BCE) is considered the period when artificial mummification began with the use of natron (mixture of sodium carbonate, hydrogen carbonate, and small quantities of chloride and sulfate), resins, and the removal of internal organs, although there is evidence for the use of embalming agents (such as heated coniferous resins, plant extracts, and gums) in the Predynastic period.^14^ The quality of mummification was highest during the New Kingdom (c. 1570 to c. 1069 BCE) and declined during the Ptolemaic and Greco-Roman periods (c. 332 BCE to c. 395 CE) until it was discontinued after the Arab conquest of Egypt in 641 CE. However, due to changes in economy, the preservation state of a mummified body of an elite member of the New Kingdom may be similar to a lower class one from the Ptolemaic period.^15^

In addition to natron salts, various aromatic materials were used to preserve the corpse and protect it from biological decomposition, such as coniferous resins and oils (e.g., pine, cedar, and juniper),^16^ gum resins (e.g., myrrh and frankincense), incense, wood, spices, herbs and flowers,^17^ vegetable oils, animal fats, and waxes.^18,19^ Knowledge of the mummification materials has been enhanced by the recent discovery of a Late Period (664–525 BCE) mummification workshop at Saqqara, where a cache of embalming pottery vessels was found with embalming instructions and names of embalming materials.^4^ Scientific analyses have also reported the use of mastic resin and bitumen,^4^ as well as Pistacia and dammar resins.^3^ Despite the frequent and rather undifferentiated use of the term “bitumen” in the context of mummification, the idea of its widespread use in embalming cannot be confirmed. Nevertheless, there is evidence of its use in the Persian period.^20^

One of the main factors affecting the preservation of mummified bodies is biodeterioration.^19,21^ After excavation, microorganisms can originate from various sources.^22^ These and their spores present in the air and on the surfaces of historical objects can proliferate only when favorable environmental conditions are met.^23,24^ Mold activity can be tracked by quantifying the secondary volatile metabolites emitted by active molds throughout their developmental stages.^25,26^ These microbial volatile organic compounds (MVOCs) can readily diffuse through porous barriers, such as textiles,^27^ and serve as indicators for detecting mold growth within the structure of historical objects.^28,29^ Molds emit approximately 150 MVOCs, and active mycelia are associated with specific compounds, including 1-octen-3-ol, 3-octanol, octanol, octanal, 1-octen-3-one, 2-octanone, 3-octanone, and octanone, collectively called eight-carbon compounds or the C_8_ complex. Additionally, heptanone, hexanone, terpenes, and sesquiterpenes, such as geosmin and isoborneol, are also often detected.^28,30^ Most studies on fungal MVOCs have focused on building materials, with limited research available on historical objects.^30−32^

The preservation of historical objects from biological degradation depends on whether microclimate parameters are kept within safe levels (*T* < 23 °C, RH < 65%).^33^ Fluctuations in temperature and humidity, as well as elevated relative humidity, exposure to or contact with water, can promote microbial growth and its proliferation.^24,34^ Climate control issues are frequently reported in museum microclimates, even in regions typically considered dry, such as Egypt,^35^ posing significant challenges. Organic substances, including proteins, fats, sugars, starch, and cellulose, facilitate the growth of molds and bacteria on mummified bodies and their covering materials (e.g., linen).^19^ The predominant species belong to actinomycetes, fungi, and bacteria.^36^ To prevent microbiological growth, synthetic and natural compounds are used as pesticides and repellents in the form of fumigants, sprays, or coatings.^37^ Approximately 90 different pesticides have been reported in museum collections,^38^ often without proper treatment documentation. Some of these are persistent and could pose health risks to staff and visitors through inhalation or dust ingestion.^39^

The condition of mummified bodies in museums and archeological sites can vary depending on the burial context, social class of the mummified bodies and quality of the mummification process, postexcavation practices, and location in heritage institution.^40^ Due to the large number of mummified bodies excavated in Egypt and stored for long periods, most mummified bodies in museum collections have undergone some form of treatment, which usually involves the use of pesticides, removal of tomb dust, or consolidation. Studies of object headspace, i.e., the space surrounding an object, confirmed that volatile pesticides can be detected, particularly in enclosed spaces.^39,41,42^ To reduce the use of potentially harmful chemicals, the Egyptian Museum in Cairo has recently introduced the use of a “pest oil”, a mixture of natural oils, as a repellent.

Ancient artifacts, including human remains, can be studied noninvasively by analyzing the volatile compounds that they emit. Volatile organic compounds (VOCs) are characterized by their high vapor pressure at room temperature^43^ and play a crucial role in the odor of objects, with their odor detection threshold (ODT) representing the lowest concentration at which a smell becomes perceptible to the human nose. The ODT is influenced by molecular properties such as shape, polarity, partial charges, and molecular mass, but also genetics.^44,45^ However, the mechanism of odor perception and the differences in the ODTs of different compounds are not well understood yet.^44^

The identification of the compounds giving a smell is crucial in the development of their olfactory profile. Gas chromatography coupled with mass spectrometry and olfactory detection (GC-MS-O) is the standard technique to determine the olfactory profile of an object.^46,47^ The olfactory detector employs a trained human “sniffer” who describes a smell in terms of quality, intensity, and hedonic tone (pleasantness), providing information on which compounds are odor-active. The coupled approach allows for the integration of chemical information from the mass spectrometer with olfactory information from the olfactometric detector, resulting in an olfactory profile. Furthermore, olfactory analysis indicates compounds at concentrations above the ODT that contribute to the perceived smell. GC-MS-O has been extensively used in food analysis,^48^ fragrance analysis,^49^ and materials testing,^50,51^ and it is now becoming popular in heritage science as highlighted by studies of smellscapes,^1^ artifacts,^46^ and materials with heritage value.^52^ An advantage of its application in the heritage science field is the possibility to obtain information on the original materials, possible degradation products, conservation materials and a clear olfactory profile. This is particularly complementary to other existing methods to account for the sensory worlds of the past, such as archival research^53,54^ and archeological evidence interpretation.^55^ Sensory analysis and GC-MS-O both provide valuable information but differ in scope. The former is used to characterize the overall odor, and the latter provides information about the individual compounds and associated smells. The combination of the two is fundamental to understand which compounds are most responsible for the perceived smell. This information is complementary as the smell of a mixture of compounds cannot be inferred from the sum of the component smells.^56^

Any study of human remains necessitates consideration of ethical implications, given their significance to the originating communities.^2^ Recent studies, e.g., forensic facial reconstruction^57^ or synthesis of the vocal sound from mummified remains,^58^ have evoked varied responses, demonstrating the need to communicate the ethical framework within which such studies are conducted. While no codified ethical guidelines exist in heritage science, researchers and professional organizations have attempted to formulate standards for the study of mummified bodies.^57^ This study supports conservation to ensure long-term accessibility, avoiding materials and techniques that could alter them in the long term, compromise future analyses, or cause loss of information.^59,60^ It involves local stakeholders in the investigation, which is essential for fostering an active research environment and promoting local awareness, thereby supporting the social sustainability of heritage.^61^ These considerations are especially relevant for ancient Egyptian mummified bodies, which were frequently subject to trade.^62^ While the international debate on the display of human remains has raised ethical conflicts and questions regarding the propriety of exhibiting corpses,^63^ increased public scrutiny prompted heritage scholars to seek a balance between respect for the human body and comprehensive understanding being provided to visitors.^64^

**Figure 1:**
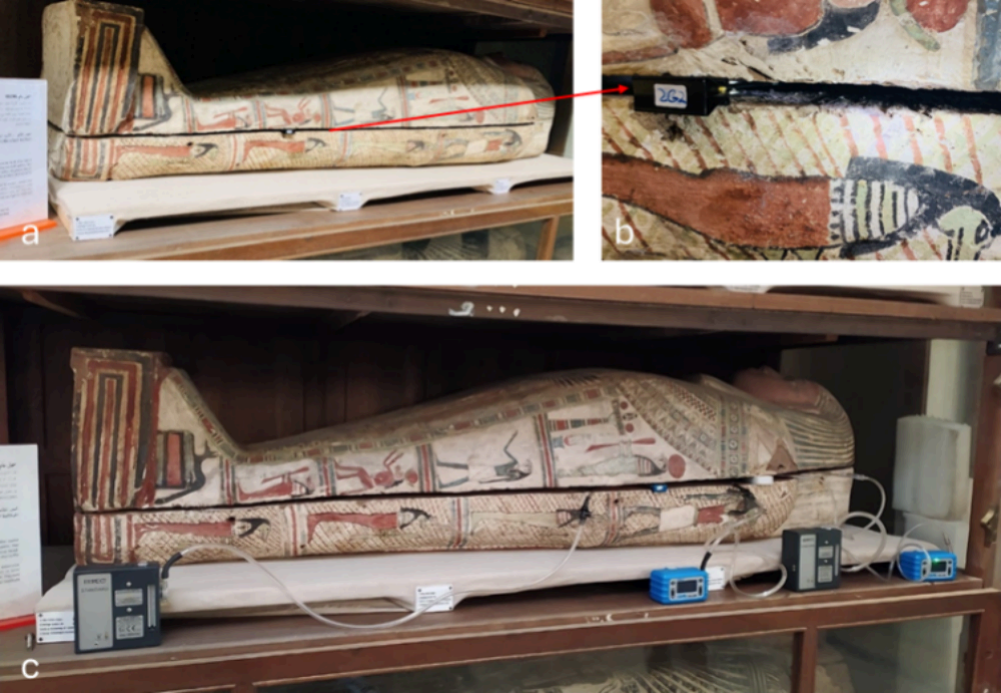
(A) Coffin with a mummified body (M7) in the display area of the Egyptian Museum in Cairo. (B) Passive sampling with SPME fiber of the headspace within the coffin. (C) Active sampling of the headspace within the coffin was performed with sorbent tubes.

This study identifies the primary components in the contemporary odor of mummified bodies. Given the diversity of mummification materials used across different time periods and social classes, the study also explores the potential for distinguishing these variations. Here, the first systematic olfactory analysis of a collection of mummified bodies, stored at the Egyptian Museum in Cairo and dating from the New Kingdom to the Roman period, is presented. We combined noninvasive headspace analysis with minimally invasive characterization of surface microorganisms. The outcomes guide conservation based on an ethical protocol for the investigation of the odor of ancient Egyptian mummified bodies (Figure 1 and Supporting Information, SI5,and Supporting Information, SI6).

## Results and Discussion

Nine mummified bodies from the Egyptian Museum in Cairo were investigated in the study. Five were located in the storage area (M1–M5), and four were located in glass and wooden display cases in the exhibition area (M6–M9). Of these, M6, M7, and M8 were displayed in a shared, compartmentalized case and M9 in a separate case. The selection presents different archeological periods, materials, and conservation histories, as well as conservation states (Table 1).

**Table 1:** Historical Information on the Mummified Bodies Based on Database Information (July 2024)

Mummified body	Location	Period	Pest treatment record	Conservation condition^a^
M1	storage			5
M2	storage	New Kingdom (c. 1539–c. 1077 BCE)		4
M3	storage	Byzantine Period (3rd–4th century CE)		5
M4	storage			3
M5	storage			0
M6	gallery	Late Period (c. 664–332 BCE)	showcase last treated with pest oil in 2021	4
M7	gallery	Late Period (c. 664–332 BCE)	showcase last treated with pest oil in 2021	6
M8	gallery	Late Period (c. 664–332 BCE)	showcase last treated with pest oil in 2021	2
M9	gallery	New Kingdom (1292–1077 BCE)	showcase last treated with pest oil in 2023	8

a The conservation condition was ranked on the scale of 0 (worst) to 8 (best) based on the condition of both the coffin and mummified body. The detailed assessment is reported in the Supporting Information, SI1 (Table 1).

**Figure 2:**
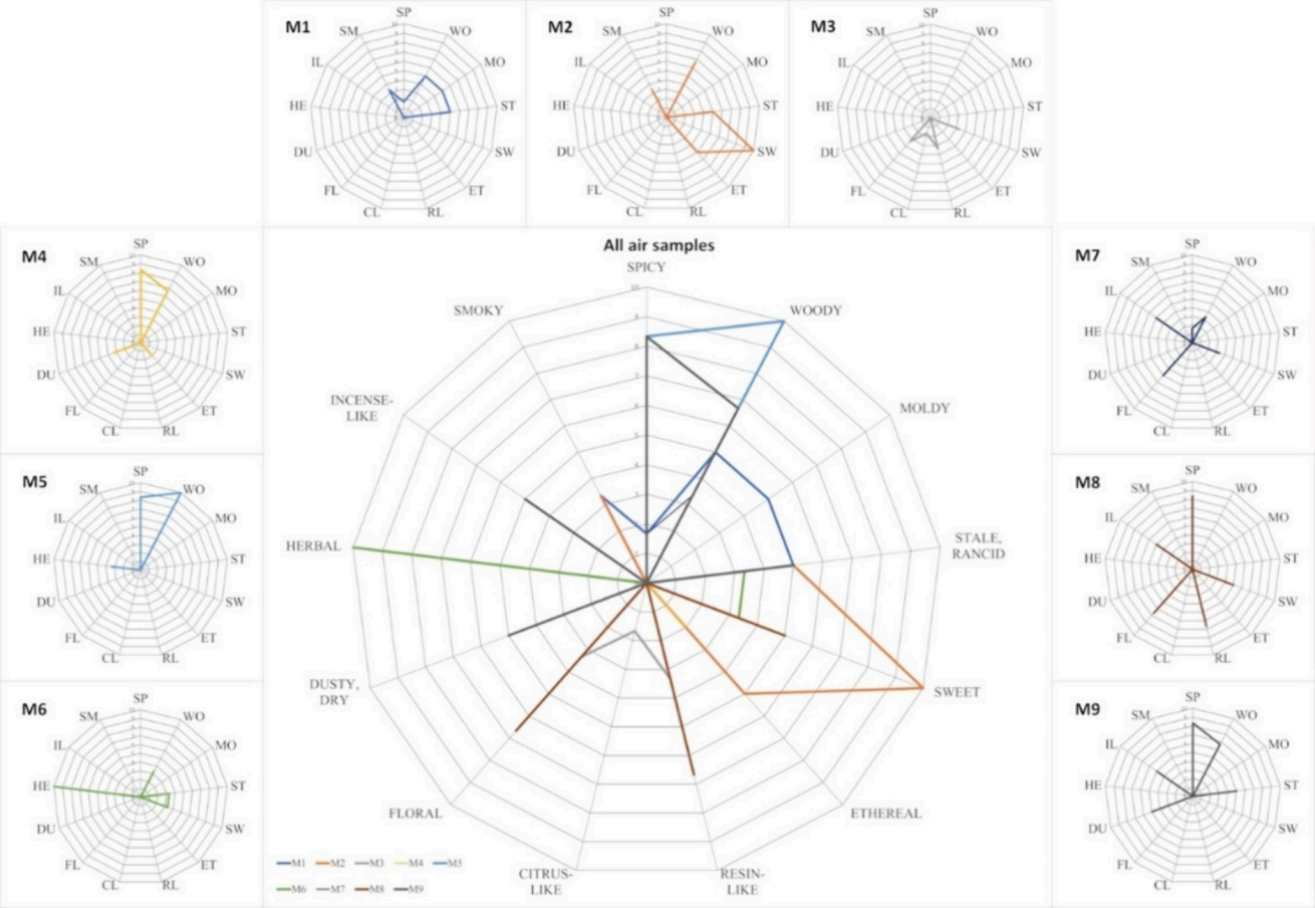
Radar plots represent odor profiles. The labels correspond to odor quality descriptors: SP = “spicy”; WO = “woody”; MO = “moldy”; ST = “stale/rancid”; SW = “sweet”; ET = “ethereal”; RL = “resin-like”; CL = “citrus-like”; FL = “floral”; DU = “dusty, dry”; HE = “herbal”; IL = “incense-like”; and SM = “smoky”. Perceived odor intensity is reported on a 0–10 scale,^65^ where 0 indicates “no odor” and 10 “very strong odor”.

During sensory analysis, we developed a set of 13 primary olfactory descriptors for the sensory assessment of the multiple case studies (Figure 2). Experts working with mummified bodies reported their smell mostly as hedonically pleasant with “balsamic” descriptors (“heavy”, “sweet”, “woody” odors). Panel evaluation confirmed this, describing the smells as “woody” (78% of the case studies), “spicy” (67%), and “sweet” (56%), followed by “incense-like” and “stale, rancid” (33% each). Other descriptors are present in fewer cases, suggesting specificity. A complete description of how the sensory analysis was performed is presented in the Supporting Information, SI1. Sensory analysis revealed distinct differences and similarities, although no significant variations in the overall intensity were observed. The average intensity of the set was “medium”, with M3 resulting in the least intense odor profile, and the hedonic tone was assessed as “slightly pleasant” on average.

**Figure 3:**
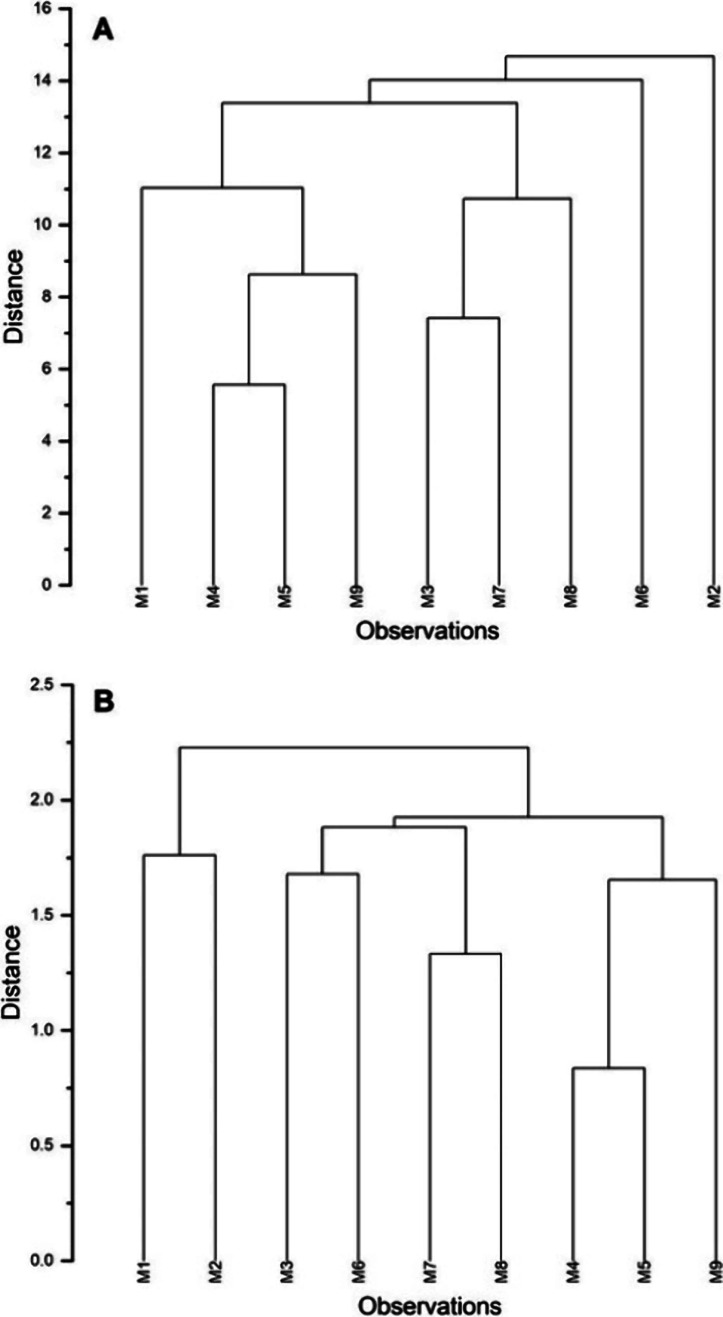
Hierarchical cluster analysis of sensory data is shown in Figure 2. (A) Non-normalized intensities, (B) normalized intensities. The data set used for hierarchical cluster analysis is available in the Supporting Information, SI4.

Hierarchical cluster analysis (Figure 3) of sensory data showed similarities, particularly the cluster of M4, M5, and M9, reflecting shared “spicy” and “woody” descriptors. All three have wood and linen in the construction, and two consist mainly of wood and linen. Since these descriptors are quite pronounced, normalization does not affect the results. Differences are revealed between individual case studies in storage, with similarities between M1 and M2 and between M4 and M5. However, given the unknown provenances of M1, M4, and M5, these results cannot be definitively linked to their history or mummification practices. There was no correlation between the conservation state and sensory profiles or intensity, as those in the worst conservation states (M4, M5, and M8) and the one in the best conservation state (M9) yielded similar data, meaning that millennia of degradation have a similar effect on the perceivable emissions.

The strong “sweet” and “herbal” descriptors make M2 and M6 stand out in Figure 3A, while the cluster of M3, M7, and M8 reflects the combination of “incense-like”, “floral”, “sweet”, and “stale, rancid”. In Figure 3B, normalized intensities increase the distances, suggesting a lower similarity. Interestingly, the mummified bodies treated with pest oil (M6, M7, M8, and M9) do not share significant similarities: M6 shows a strong “herbal” descriptor, while M7 and M8 display pronounced “floral” descriptors, which are not shared by the nontreated ones, except by M3 to a small extent. Sensory analysis of the pest oil indicates that five descriptors out of 13 are in common with the ones used for the mummified bodies: “spicy”, “sweet”, “ethereal”, “citrus-like”, and “floral” (Supporting Information, SI1, Figure 38). This suggests that the overall smell of the mummified bodies as perceived by the panelists is influenced only to a small extent by the treatment oil.

**Figure 4:**
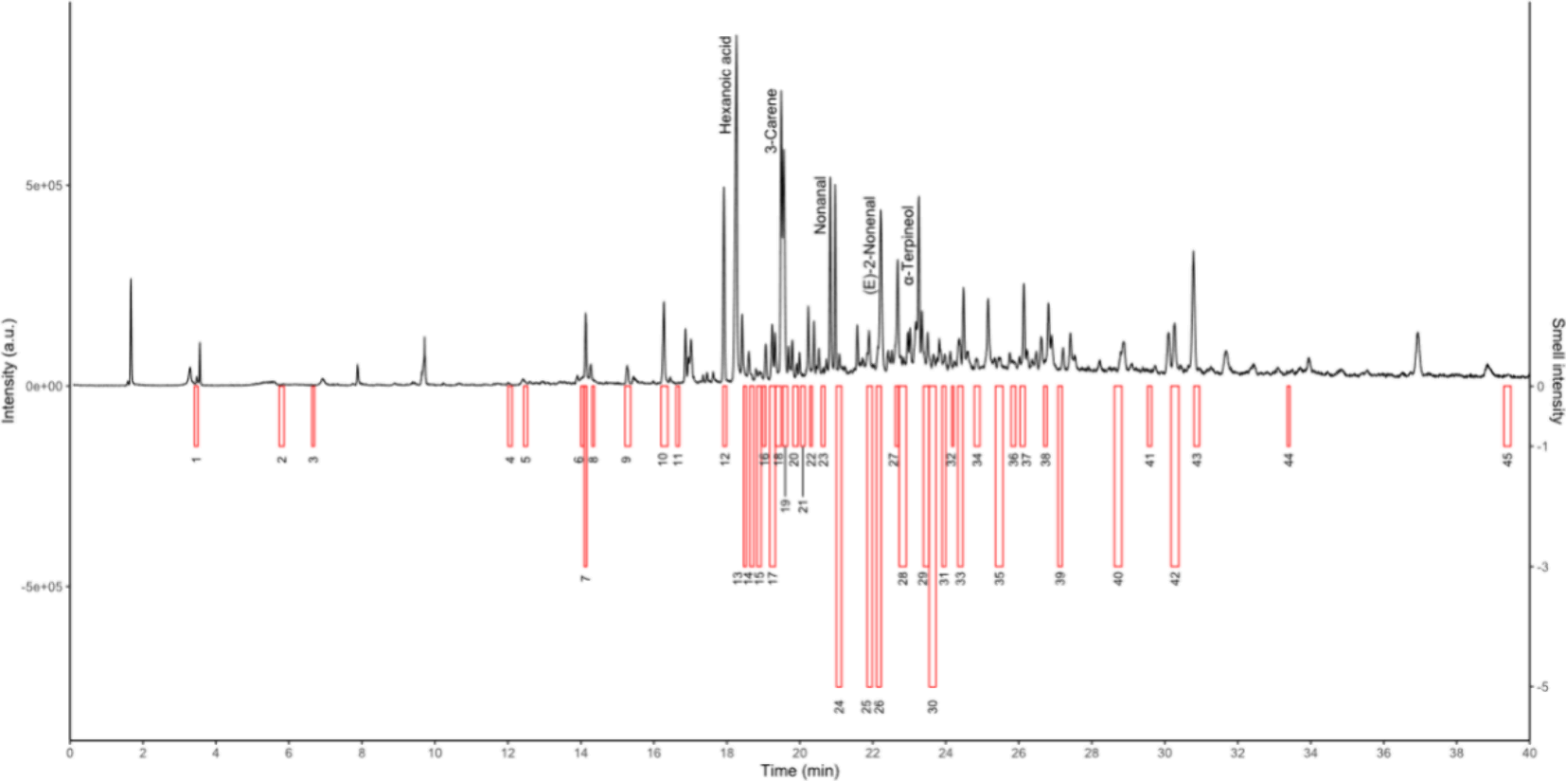
Comparison of the MS chromatogram (black) with the olfactogram (red) for sample M8 (subsample 4) highlighting the differences between the two methods of detection. For numeric labels refer to the Supporting Information, SI3.

The results of the analysis with GC-MS-O are visualized in plots combining chromatograms and olfactograms. These provide a clear overview of the number of odor-active compounds, showing a rich olfactory profile. The number of peaks in the chromatogram and in the olfactogram may differ based on whether the compounds detected by MS are odor-active. The intensities of the MS signal and the perceived smell are not always proportional, as compounds with a low odor threshold might be perceived as high-intensity smells but be detected with low intensity by the MS. An example is M8 (Figure 4) where fenchol was detected with medium-low intensity at 21.9 min in the chromatogram but was perceived as strong with olfactometric detection (Figure 4: smell label 26). All of the recorded plots are available in the Supporting Information, SI1.

The sensory analysis of M8 resulted in “spicy” (intensity: 8), “resin-like” (7), “floral” (7), “sweet” (5), and “incense-like” (5). However, GC-MS-O identified predominantly “cheese”, “wax”, “floral”, “herbal”, and “green”, highlighting some overlap but no direct correspondence with sensory findings. This discrepancy underscores that the perception of an odor mixture does not necessarily align with the perception of its individual odorants. In contrast, a correlation between sensory and chemical analyses is evident in M6. During panel evaluation, caryophyllene was only identified in this case study with its characteristic “tea-like” smell (intensity: 10), categorized as “herbal” when in the selection of the 13 descriptors used in Figure 2. With GC-MS-O analysis, caryophyllene was consistently described as “woody” and “herbal” during olfactory analysis and identified as caryophyllene with MS detection, confirming the “tea-like” characteristic resulting from sensory analysis.

Among the identified VOCs, most can be assigned to four categories based on their origin: (i) materials used during the mummification process and their degradation products; (ii) MVOCs resulting from microbiological activity; (iii) pest oil used for conservation; and (iv) synthetic pesticides.

In contrast to sensory analysis, GC-MS-O shows notable differences in both the intensity and quantity of compounds, both between case studies and at different locations. A selection of the identified compounds based on the intensity of the smell detected during the GC-MS-O analysis, their repeatability in replicate analyses and their relevance to interpretation, is reported in Table 2. An exception is the group of synthetic pesticides, in which odorless compounds are included for health and safety reasons. Their presence in Table 2 allows direct comparison of the mummified bodies treated with the pest oil and those treated with pesticides.

**Table 2:**
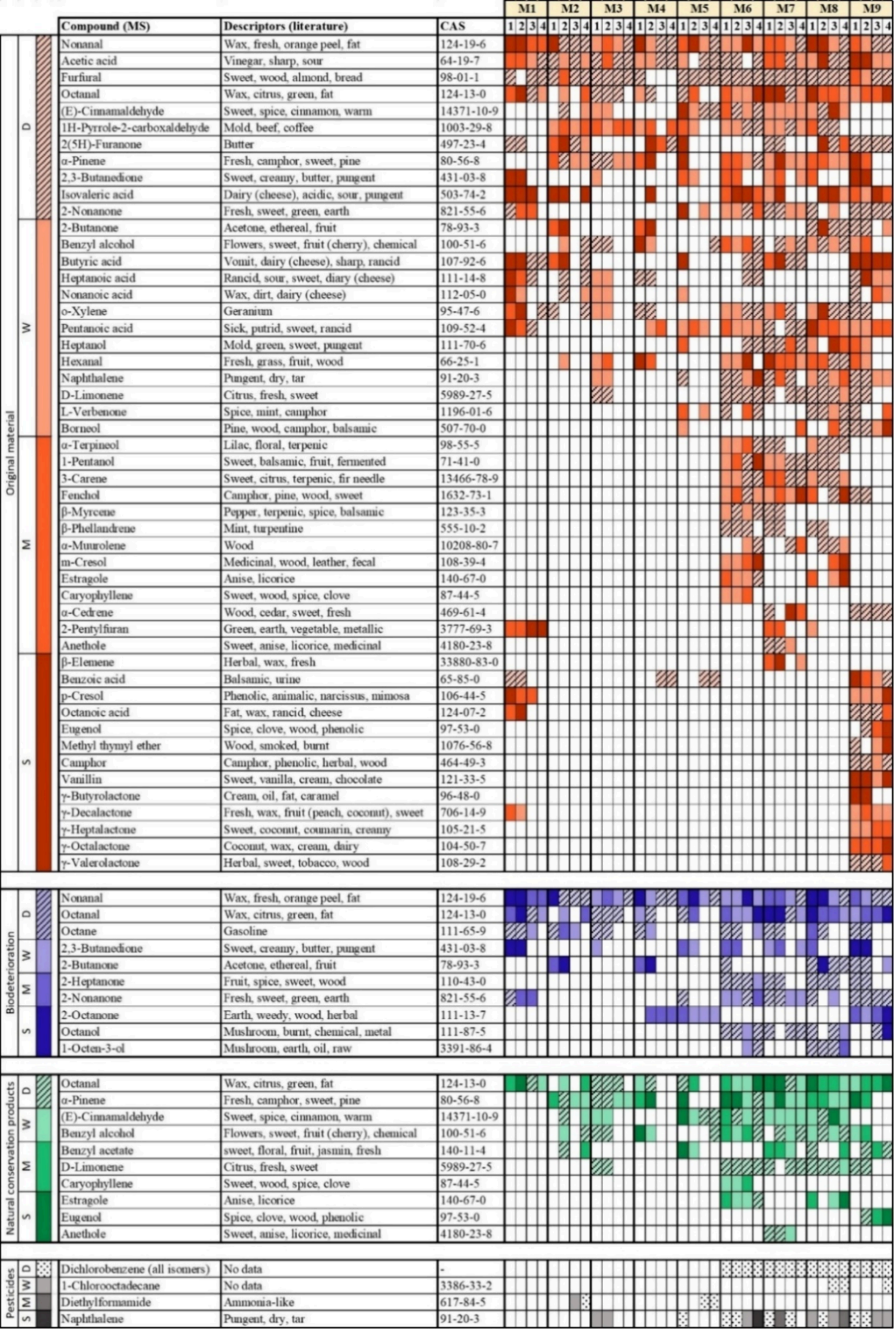
List of Compounds Identified in the Replicate Analyses and Correlated to the Presence of Original Mummification Materials, Conservation Treatments, or Microbiological Activity^a^

a Each identified chemical compound is associated with its olfactory descriptors.^66−68^ Only smells recorded at least twice per case study or once in at least two case studies are reported as significant (the full list of identifications for each of the replicates is shown in the Supporting Information, SI2). Color intensity is related to the smell intensity. 1,2, refers to sample replicates analyzed at the Krakow University of Economics, and 3,4, refers to samples analyzed at the University of Ljubljana. More details of the chemical analysis are present in the Supporting Information, SI2, and Supporting Information, SI3. The intensity is defined as S = strong, M = medium, W = weak, and D = only detected by MS, not smelled by a sniffer.

The compounds in the category “pest oil used for conservation” result from the chemical analysis of the pest oil currently used at the Egyptian museum (green cells in Table 2). These, combined with museum records, indicated the use of a mixture of clove oil, camphor oil, peppermint extract, basil oil, lemon oil, orange oil, and cinnamon oil in treating the display cases. In addition to these, common compounds found in essential oils for conservation are added: octanol (orange oil), eugenol (clove, cinnamon, basil oil), and α-pinene (pine, orange, and juniper oil).

Table 2 illustrates that many odor-active compounds were exclusively or more intensely detected in case studies from the exhibition area compared to those from storage. This variety may result from the better preservation state (M8 being an exception) but also from the fact that the displayed mummified bodies are enclosed in display cases, allowing volatiles to accumulate. In the original materials class, acetic acid and furfural, indicators of wood and cellulosic material degradation, were detected in all samples. 2(5*H*)-Furanone and vanillin, lignin degradation products, were also identified, with 2(5*H*)-furanone in most samples and vanillin only in M9. Similar compounds were identified in M2, M3, and M4. M1 and M5 did not exhibit any similarities to the other case studies. M1 showed a higher overall intensity of detectable smells, with almost all volatiles identified by MS also being detected by the sniffers.

No unique compounds were found exclusively in the storage area, while the display area had a greater variety, mainly terpenoids, lactones, and phenolic compounds. Of the terpenoids, α-pinene was predominant, with d-limonene, l-verbenone, and borneol being more common in the display area yet also present in the storage area. Terpenoids suggest the use of plant products such as juniper oil, myrrh, and frankincense, during mummification, as documented.^15^

l-Verbenone is characteristic of coniferous plants, specifically pine and cedar,^16^ while borneol is derived from resins or as an oxidation product of camphor. All other identified terpenoids belong to the monoterpene and sesquiterpene groups and are detected only in the display area. They indicate the use of cedar or pine resin, gum resins like myrrh and frankincense, and other plants such as thyme, lavender, and eucalyptus. Due to the presence of these compounds in various plant sources, unequivocal assignation is not possible.

Furthermore, lactones were detected uniquely in M9 and clearly detected by almost all sniffers likely due to low levels of ODTs and distinctive sweet, fruity smells, particularly, γ-decalactone and γ-octalactone with ODTs of 0.005–0.01 ppm and 0.01–0.02 ppm, respectively. These may be derived from plant extracts, oils, or resins used in embalming.

The detection of MVOCs (Table 2) indicates active microbiological activity that may originate from lipid degradation of essential oils, animal fats, or the human remains themselves.^69^ However, MVOCs are not species-specific and thus cannot indicate which species, among those identified via microbiological analyses, were active or dormant. The microbiological analysis (Supporting Information, SI1, Tables S4–S6) revealed a variety of species: *Aspergillus niger*, *Penicillum chrysogenum*, *A. flavus*, *Cladosporium cladosporioides*, *Rhizopus oryzae*, *Bacillus subtilis*, and *B. pumilus* being the dominant species. The latter are common environmental bacteria that contribute to the biodeterioration of natural polymers.^70,71^ Other bacteria, such as *Cytobacillus oceanisediminis* (previously isolated from Egyptian historical sites^72^) and *Priestia megaterium* (found on Tehran museum objects^73^), were also identified. Dormant species and spores were found, although the results can be affected by microorganism interactions. Namely, various *Bacillus* species, e.g., *B. velezensis* and *B. pumilus* identified here, can eliminate other microorganisms to the extent of becoming undetectable. If spores of these species were present, then the presence of other (active) microorganisms may not be detectable. Despite the presence of bacteria and mold, air concentrations do not exceed health and safety limits, and the fungal species observed are commonly reported in museum studies, including mummified bodies.^19,22,74^ The MVOCs emitted are typically short chain aldehydes, ketones, and alcohols, and given their presence in diverse sources, it is not possible to unequivocally categorize them. For example, octanal and nonanal can originate from plant oils (orange and citrus oil), microbiological activity, lipid oxidation, or degradation of human remains.

Synthetic pesticides (black cells in Table 2), detectable in the display area, were unexpected as no conservation reports mentioned their use. Their identification during preliminary SPME analysis (Supporting Information, SI1) required a specific ethical and safety assessment before GC-MS-O analysis (Supporting Information, SI6). The display cases of M6, M7, M8, and M9 were all treated with pest oil in the recent past (Table 1) and with synthetic pesticides according to our results, which could contribute to their state of preservation. These pesticides were mostly odorless, which explains why most were detected by MS, but did not contribute to the smell.

**Figure 5:**
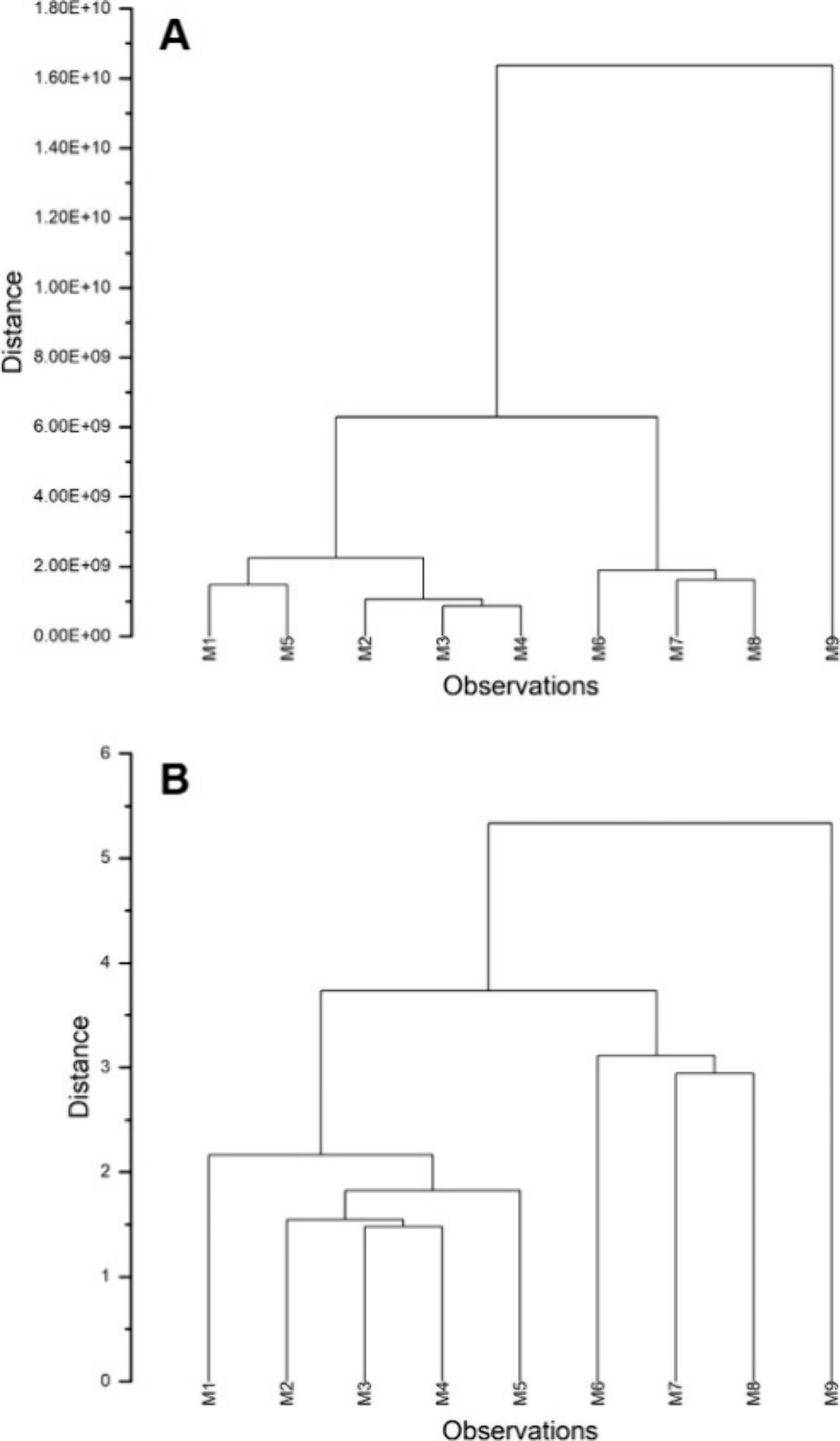
Hierarchical cluster analysis of average peak areas for the selection of odor-active compounds emitted (Table 2). (A) Non-normalized intensities, (B) normalized intensities. The data set used for hierarchical cluster analysis is available on the Supporting Information, SI4.

A hierarchical cluster analysis of the average (four analyses) integrated peak areas for the odor-active compounds (Table 2) shows clearer distinctions than Figure 4, almost regardless of whether non-normalized or normalized data are used (Figure 5). Since only odor-active compounds were selected, synthetic pesticides were excluded. The hierarchical cluster analysis of the chromatographic data clearly separates the case studies from the storage and display areas. Coming from the storage area, M1–M5 are generally characterized by less intensive peaks, and they do separate from the rest even in the normalized plot. Unlike the exhibition room case studies, normalization does affect how these are clustered: M1 and M5 are grouped together in the non-normalized cluster plot, which make sense given that the textile wrappings of both are highly carbonized. M3 and M4 are both poorly equipped, with only some textile and a terracotta (M3) and a wooden coffin (M4). M2, on the other hand, has richer decoration and material composition. Among all, M9 clearly stands out with more compounds often in higher concentrations. Given that M9 is in the best conservation condition, even if it is one of the oldest of the dated ones, it is possible that many compounds (e.g., lactones) reflect the presence of authentic mummification materials, rather than pest oil. Although no information about the deceased is available, the coffin is decorated with a gilded mask, suggesting an elevated social status, which could be reflected in a better quality of mummification and therefore more remaining odor-active compounds.^75^

There are similarities in the chemical analysis between M1 and M9, related to the presence and absence of benzoic acid, octanoic acid, p-cresol, (*E*)-cinnamaldehyde, and 1*H*-pyrrole-2-carboxaldehyde. These suggested that similar mummification practices, storage conditions, or similar degradation or conservation processes may have been used for M1 and M9. However, hierarchical cluster analysis does not highlight these similarities.

Differences in period (M6, M7, and M8 from the Late Period, and M2 and M9 from the New Kingdom) did not reflect a significant variation of emissions, with only lactones differentiating M9 from the case studies from the Late Period. Despite M2 and M9 both being from the New Kingdom, they show no similarity in either sensory or chemical analysis, nor do they cluster together in Figure 5. In contrast, M6, M7, and M8 exhibited strong sensory and chemical similarities, as shown in the cluster analysis (Figure 5). This is likely influenced by the use of similar Late Period mummification materials. However, they could be influenced by the use of pest oil or potential cross-contamination from the shared display case. The use of pest oil may explain the presence of (*E*)-cinnamaldehyde and octanol in most case studies, although treatment records only exist for the display cases of M6, M7, M8, and M9. In addition, the results from sensory analysis suggest that the pest oil has little influence in the overall smell, confirming the that it is mainly influenced by the original materials and their degradation products, and only to a smaller extent by the treatment oil, thus supporting the authenticity of the results obtained via both sensory and chemical analyses.

## Conclusions

The robust approach to sensory and olfactory analysis applied to nine mummified bodies yielded a complex data set offering insights into the mummification practices, as well as conservation history and preservation states. The integration of olfactory analysis with traditional and well-established chemical techniques gave the possibility to develop a new approach focusing on the compounds actually contributing to the current smell of the mummified bodies.

By combining sensory, chemical, microbiological, and historical research, this study developed a novel, nondestructive approach to studying ancient remains, reflecting the complexity of mummification practices, diversity of materials, and divergent conservation histories. Working with local stakeholders, with first-hand experience of the mummified bodies through professional practice, enriched the collected vocabulary (e.g., by suggesting smell descriptions no other analyst had used, indicating a perceptual lens differing from the European analysts). These data sets improve our capacity to describe the olfactory qualities of the mummified bodies and build cocreated, multicultural vocabularies, which enable new interpretations of the sensory past.

The detected volatiles fell into four categories: those originating from archeological materials, conservation products, synthetic pesticides, and biodeterioration products, although some compounds cannot be unequivocally categorized. The results revealed that the exhibited mummified bodies show a greater variety and higher concentration of compounds compared to those in the storage, likely due to the accumulation of volatiles in the display cases. Notably, phenolic compounds, lactones, and numerous terpenoids were detected only in the display area.

The results also revealed close similarities between mummified bodies from the Late Period, indicating that with a larger set with more detailed information on the mummified bodies, it may be possible to differentiate by the period (or at least by the mummification practice) based on chemical and olfactory profiles and to achieve a better understanding of the different practices.

This highly interdisciplinary study provides valuable scientific data that could help in the development of novel museum practices:

(i) The analyses directly contribute to improving conservation by identifying pesticides or other organic toxic compounds that could harm museum workers. Upon identification, specific handling guidelines can be followed or the materials can be placed in separate display cases.

(ii) The olfactory analyses inform us of the current smell emitted by the materials. This olfactory heritage should be preserved as an integral part of the mummified body’s significance, and to this end appropriate preservation strategies are essential.

(iii) Based on the findings, it is possible to practically intervene by storing the mummified bodies in display cases rather than in loose wrapping.

(iv) More broadly, the preservation of the olfactory heritage of case studies requires systematic archiving of the collected data, chemical and olfactory, in repositories, ensuring availability for future research and interpretation.

## Supporting Information Available

Description of the mummification practice, historical information, health and safety risk analysis, experimental section, microbiological analysis results, chromatograms and olfactograms, and radar plot for the pest oil odor profile (PDF)
GC-MC-O identification data (XLSX)
GC-MC-O identification data per each replicate (XLSX)
Hierarchical cluster analysis dataset (XLSX)
Informed consent for participation in sensory analysis (PDF)
Informed consent for participation in olfactory analysis (PDF)
