# Supplementary material for: Ancient Egyptian Mummified Bodies: Cross-Disciplinary Analysis of Their Smell
Source: J Am Chem Soc. 2025 Feb 13;147(8):6633–43. doi: 10.1021/jacs.4c15769 (PMC11869298; doi:10.1021/jacs.4c15769)
Supplement: Supplementary file 1 — ja4c15769_si_001.pdf [file ja4c15769_si_001.pdf]

Ancient Egyptian Mummified Bodies: Cross-disciplinary Analysis of Their Smell

Emma Paolin[a], Cecilia Bembibre[b], Fabiana Di Gianvincenzo[a], Julio Cesar Torres-Elguera[c], Randa Deraz[a], Ida Kraševec[a], Ahmed Abdellah[d], Asmaa Ahmed[d], Irena Kralj Cigić[a], Abdelrazek Elnaggar[a,e], Ali Abdelhalim[d,e], Tomasz Sawoszczuk[c], Matija Strlič[a,b]*

[a] Heritage Science Laboratory Ljubljana, Faculty of Chemistry and Chemical Technology, University of Ljubljana, Večna pot 113, Ljubljana 1000, Slovenia

[b] Institute for Sustainable Heritage, University College London, 14 Upper Woburn Place, London WC1H 0NN, U.K.

[c] Department of Microbiology, Institute of Quality Sciences and Product Management, Krakow University of Economics, Henryka Sienkiewicza 4, Krakow 30 - 033, Poland

[d] The Egyptian Museum in Cairo, Cairo 4272083, Egypt

[e] Faculty of Archaeology, Ain Shams University, Abbasiya Cairo 11566, Egypt

Supplementary Information 1

Table of Contents

1. The mummification practice 2
2. Mummified bodies: historical information 2
3. Health and safety risk analysis 4
4. Experimental section 4
   4.1 Sensory analysis 4
   4.2 Gas chromatography coupled to mass spectrometry and olfactometry 6
   4.3 Microbiological analysis 6
   4.4 Data analysis 7
5. Microbiological analysis: results 7
6. GC-MS-O chromatograms/olfactograms 9
7. Radar plot for the pest oil odor profile 29
8. References 29

1. The mummification practice

This section provides a brief overview of the process of mummification, for further information, the reader should consult the cited references.

High-quality mummification was achieved through several processes. The first step involved desiccation of the body tissues using natron salts[1], a desiccant known in ancient Egyptian history and preferred over sea salt due to its effectiveness in quickly drying out the body and saponifying fatty tissue. This was followed by evisceration, the removal of lungs, liver and stomach, and excerebration, the removal of the brain. The cavities in various parts of the body were filled with packing materials, such as crushed myrrh, cinnamon, frankincense, sawdust mixed with resin, cassia[2].

Certain oils, believed to have protective, purifying, and spiritually uplifting properties, were used due to their aromatic qualities and significance in rituals and burial practices. Examples include cedar, juniper, and pine oils[3]. The body was then covered with oils and ornaments, and molten resin was applied to the skin and/or poured into the skull and other body cavities, to prevent the growth of bacteria and act as a deodorant. Finally, the body was wrapped in resin-soaked linen bandages and buried in a sealed burial environment to prevent degradation[4]. Additionally, the mouth, eyes, and ears were typically covered with wax, and the hair was often dyed[2].

The removal of soft tissue was a fundamental step to promote the preservation of the body because it is subject to rapid decomposition by insects and microorganisms. In another, less costly mummification technique, the body was not disemboweled. Instead, cedarwood oil was injected into the anus, which was then sealed. After treating the body with natron and allowing it to dry[5], the oil was drained, and the decomposed intestines were removed with the oil. During the Ptolemaic period, variations included either returning the viscera to the body or wrapping the body in balls of resin-soaked linen, mud, melted resin, or bitumen[4].

2. Mummified bodies: historical information

M1 (ID: N 305): Fully wrapped mummified body without coffin in fragile burned and blackened linen. Materials of the mummified body: carbonized textile, wooden painted mask, resin*.The face is covered with a gilded mask. It is stored on a wooden stand and covered with Tyvek.

M2 (ID: ID 179, N 281): Anthropoid coffin painted black with unwrapped mummified body inside. The coffin has no inscription and the state of preservation is poor. The materials of the coffin are wood, pigment layers, gypsum*, textile, clay*, black paint or resin+. The wooden coffin is covered with one Tyvek and one PVC layer. It is stored in a large wooden box without a lid, placed on the ground. With Tyvek® wrapping followed by PVC wrapping.

M3 (ID: CG7216, SR 4/12008, N 302): A pottery coffin with the mummified body of a child. State of preservation is poor and the mummified body is not wrapped, some textile fragments are enclosed in the coffin. Some parts of the body are crushed, the head is partially smashed and there are scraps of linen in the coffin.

M4 (ID: TR 12/10/37/1, N 226): Wrapped mummified body covered with linen, probably, inside a base of wooden coffin. Poor state of preservation, eroded from the side. Wood of the coffin disintegrating to powder in some points. No protective layer on the mummified body.

M5 (ID: N 343): Mummified body covered with cartonnage with small pottery jars lying beside. The body is partially wrapped (original wrapping made by carbonized textile, resin*), but smashed from the sides. The materials of the coffin are wood, cartonnage, pigment layers, gypsum*, textile, clay*. The state of preservation is very poor. It is covered with a layer of PVC with a thin wooden protective cover and placed on a wooden shelf.

M6 (ID: JE 66783): Mummified body covered in textile inside one of the coffins of El-Hibe. The coffin is displayed on a linen-covered wooden stand designed specifically for El-Hibe coffins. The materials of the coffin are wood, pigment layers, gypsum*, textile, clay*. The preservation state of the body in the coffin could not be assessed. The materials used for conservation are Paraloid 72, Japanese tissue paper, Klucel G, and microballoon bulking agent. It is stored in a showcase (wood, glass, textile) with a label (paper, acrylic, printing ink).

M7 (ID: JE 66786): Wrapped mummified body inside one of the coffins of El-Hibe. It was displayed on a linen-covered wooden stand designed specifically for El-Hibe coffins. The materials of the coffin are wood, pigment layers, gypsum*, textile, clay*. Preservation state is good, except for the head that is separated from the body. The materials used for conservation are Paraloid 72, Japanese tissue paper, Klucel G, microballoon bulking agent. It is stored in a showcase (wood, glass, textile) with a label (paper, acrylic, printing ink).

M8 (ID: JE 66790): Wrapped mummified body inside one of the coffins of El-Hibe. Very poor preservation state as parts of the mummified body are crushed and turned into powder, with burned and blackened linen. The materials of the coffin are wood, pigment layers, gypsum*, textile, clay*. The materials used for conservation are Paraloid 72, Japanese tissue paper, Klucel G, microballoon bulking agent. It is stored in a showcase (wood, glass, textile) with a label (paper, acrylic, printing ink).

M9 (ID: Index: 11328, (Temporary 6/9/16/4)): Wrapped mummified body from the 19th-20th dynasties inside an anthropoid coffin with gilded face and spiral lines on the chin, with a blue wig. The eyes of the coffin had been inlaid. The wood is carefully shaped, and there are five vertical lines of hieroglyphic inscriptions. The mummified body is decorated with the four sons of Horus and is gilded on each side. There is a pendant seal on the cloth. Excavation: Northern upper Egypt - Akhmim (IPU, Khent-min, khmmis, panopolis). The materials of the coffin are wood, pigment layers, gypsum, gilding. It is stored in a showcase (wood, glass, textile) with a label (paper, acrylic, printing ink).

Identification *likely/+possible, based on visual inspection.

Supplementary Table 1. Assessment of the conservation state of the mummified bodies.

	**Coffin condition**		**Mummified body condition**		
**Mummified body**	Coffin<br><br>2 (all parts exist)<br>1 (partially missing)<br>0 (largely missing)	Coffin materials<br><br>2 (good state)<br>1 (moderate state)<br>0 (bad state)	State of original wrapping<br><br>2 (good state)<br>1 (partially torn)<br>0 (largely torn)	Body parts<br><br>2 (good state)<br>1 (partially damaged)<br>0 (largely damaged)	Condition score
**M1**	0	2	1	2	5
**M2**	2	2	0	0	4
**M3**	2	2	0<br>(There are textile scraps in the coffin, the body may have been wrapped before)	1	5
**M4**	1<br>(Missing parts)	0<br>(Coffin base is fragile and powdery)	1	1	3
**M5**	0	0	0	0	0
**M6**	2	0<br>(Wood of the coffin is very fragile)	Not accessible	Not accessible	4 (weighted)
**M7**	2	2	1	1<br>(Parts separated)	6
**M8**	1<br>(The coffin base is missing)	1	0	0	2
**M9**	2	2	2	2	8

The state of preservation is classified on a scale from 0 (coffin and body are largely damaged) to 8 (coffin and body are well preserved). The assessment was carried out on the basis of visual inspection of (1) the coffins: whether all parts were are present or whether a coffin lid or base is missing, and (2) the condition of the body: whether the original wrapping is still in its original condition, whether the mummy body parts are completely, partially, or not damaged.

3. Health and safety risk analysis

Preliminary SPME screening showed that some potentially harmful compounds are present in the headspace of the mummified bodies, such as the chlorinated compounds 1,2-dichlorobenzene and 1,4-dichlorobenzene (that are used as fumigants against moths and mold[6]), and the broad-spectrum organophosphate pesticide Chlorpyrifos.
At a normal respiratory rate of 12-20 breaths per minute[7] and 400-500 mL of air per breath[8], in an environment with a substance concentration at the short-term exposure limit (STEL)[9], the maximum permitted inhaled dose is 46 mg for 1,2-dichlorobenzene, 9 mg for 1,4-dichlorobenzene and 0.09 mg for Chlorpyrifos (calculated as a product of STEL, exposure time of 15 min, respiratory rate and breath volume).
The mass of analytes usually injected in a GC chromatographic system is of the order of magnitude of 1 ng[10], while a typical peak of limonene that can be smelled during olfactory analysis requires the injection of about 1 µL of a 0.1 mg/L solution, resulting in no more than 0.1 ng of limonene inhaled. It should also be highlighted that the injection of all samples in split mode (see Methods section in the main text of the article) further reduces the amount of each analyte in the air inhaled by the sniffer.
These calculations show that the amount of compounds, inhaled by the sniffer during a GC-MS-olfactory analysis, is 4-7 orders of magnitude smaller than the exposure limits[9] and therefore represents a low risk to the sniffer in the course of an analysis. Considering that most of the STEL available in the EH40/2005 Workplace exposure limits[9] are in the same or higher range than the value for Chlorpyrifos, this conclusion can be applied to GC-MS-O analysis in general.

Supplementary Table 3. Summary of the risk assessment for the synthetic pesticides detected in the emissions of the mummified bodies.

Substance	Adverse health effects	Short-term exposure limit (15-minute reference period)[9]	Max permitted inhaled dose in 15 min
1,2-Dichlorobenzene	Eye and respiratory irritation at high concentrations, no concern for the carcinogenic potential[11]	50 ppm or 306 mg/m3	46 mg
1,4-Dichlorobenzene	Eye and respiratory irritation at high concentrations, harmful effects on the liver, skin, and central nervous system at long-term exposure[6]	10 ppm or 60 mg/m3	9 mg
Chlorpyrifos	nausea, dizziness, confusion, respiratory paralysis at high concentrations, developmental neurotoxicity[12]	0.6 mg/m3	0.09 mg

4. Experimental section

Ethical assessment. The ethical challenges required a two-sided approach: (i) clear justification and needs assessment in collaboration with the Museum authorities, and (ii) development of consensus questionnaires and health impact assessments for sensory panelists and sniffers (SI5, SI6).

4.1 Sensory analysis

Odor assessment panel training. The sensory tests were conducted with volunteers from the University of Ljubljana (UL), University College London (UCL) and the Egyptian Museum in Cairo (EMC), exhibiting no known illness at the time of examination. The panel consisted of 3 women and 5 men, aged 25 to 50 years. In several sessions, most of the panel was trained in the assessment of c. 25 to 65 selected odorants at different concentrations according to their odor qualities. This enabled the calibration of the naming of these odorants,

reaching a common language. While the panelists from UL and UCL had no prior experience with the smell of mummified bodies or mummification materials (such as balms, oils and resin), apart from the referents listed in SI1 table 2, workers form EMC had a broad experience working with mummified bodies and therefore were familiar with a range of their characteristic smell. All the volunteers took part in the training with the main compounds associated with mummification materials, however the three EMC assessors showed a higher familiarity with the smellscapes of the samples through their professional experience in curation and conservation. The odor referents presented to the panelists included a wide spectrum of smells as proposed by the Field of odors model[13] and a selection of sample-relevant odorants (SI1 Tab. 2) identified through literature review[14-18]. Including panelists with extensive knowledge of the sample odors enriched the discussions, allowing for the selection of key olfactory descriptors and the incorporation of additional descriptors beyond the initial referents. In fact, in addition to the referents in SI1 Tab.2, odor referents with the quality descriptors 'rotten', 'mushroom', 'damp', 'musk', 'mold' were shared as potentially relevant to characterize aspects of biodeterioration in the samples.

Supplementary Table 2. Sample-relevant odor referents used during the panel training.

Referent number	Referent	Function	CAS number or provenance	Odor description
1	D-Limonene 10%	Olfactory function assessment	138-86-3	Citrus orange fresh sweet
2	α-Pinene	Sample-specific training	7785-70-8	Pine, conifer-like
3	Pentanoic acid 1%	Olfactory function assessment	109-52-4	Sickening putrid acidic sweaty rancid
4	Cypress oil	Sample-specific training	n/a	Fresh pine woody earthy olibanum dry spicy cedar
5	Cedar oil (Virginia)	Sample-specific training	n/a	Woody/dry, typical cedar note, pencil note
6	Dammar Resin	Sample-specific training	L. Cornelissen & Son, London, UK	Lemony, light, ethereal
7	coumarin	Sample-specific training	91-64-5	Sweet hay tonka new mown hay
8	Olibanum (Arabian incense)	Sample-specific training	n/a	Lemony, green, terpenic
9	Beeswax	Sample-specific training	n/a	Sweet honey balsam waxy hay tobacco spice
10	Bitumen	Sample-specific training	Archaeological material collection at University of Ljubljana	Petrol, burnt wood, asphalt, seaweed

Sensory analysis. An average of 20 L of the headspace around the sample was collected in polyethyleneterephthalate Nalophan NA gas sampling bags, conforming to EU EN 13725:2022 for the sampling of odor samples (Olfasense, Kiel, Germany) using a TinypumpX pump (Flextailgear, Shanghai, China). The panel performed analysis in a neutral environment at a temperature of 28 °C and RH of 60%. Odor intensity, hedonic tone and quality were individually assessed for each sample and later discussed by the panel to reach consensus.

The sensory analyses were conducted in two sessions, with the samples being presented in 10-30 L Nalophan NA gas sampling bags, considering the optimum sniff as an inhalation of a minimum of 0.45 s at a rate of 30 L/min[19]).

Following an initial exploration of reported materials linked to embalming materials and biodeterioration[14,20], cross-referenced with odorant databases[21-23] a curated set of 13 descriptors was presented to evaluators to consider during the sensory panel. Through in-situ panel discussions, additional descriptors specific to particular case studies or subsets thereof were identified and collectively agreed upon, an approach found effective by previous analyses of historic sensory qualities[24]. Panelists were asked to assess each sample individually, naming olfactory attributes and rating their intensity for each attribute on a given scale of 1 to 6 corresponding to low (1-2), medium (3-4) and high (5-6) odor. In a second session, panelists discussed their individual impressions and reached consensus on a set of 3-5 odor descriptors for each sample, with related intensity. The overall intensity for each sample was assessed on a scale of 1 to 10 and hedonic tone was rated on a +4 to −4 scale. Interpretation of sensory experiences of historic objects is limited by several factors, such as changes in the original smells stemming from aging of the material source; human interpretation of olfactory experiences being

attached to period-dependent perceptual models[25], and the limitations of the analytical methods when working with unique samples.

4.2 Gas chromatography coupled to mass spectrometry and olfactometry

SPME analysis. Volatile compounds emitted from nine mummified bodies are sampled with solid phase microextraction (SPME) fiber divinylbenzene-carboxen-poly(dimethylsiloxane) (DVB/CAR/PDMS) type (57348-U; Supelco-Sigma Aldrich) for 24 h. SPME fibers are analyzed with GC-MS/MS (TRACE 1300 GC, TSQ 9000, Thermo Fisher Scientific, Waltham, MA, USA) at the following conditions: HP-5MS column (30 m × 0.25 mm/0.25 µm, Agilent J&W, Folsom, CA, USA); He flow at 1.0 mL/min; splitless injection; inlet temperature 240 °C; temperature program (40 °C, 2 min; 220 °C, 10 °C/min; 250 °C, 20 °C/min); ion source temperature 280 °C; MS transfer temperature 280 °C; MS was operated in TIC mode in the range *m/z* 43–400.

Gas chromatography coupled to mass spectrometry and olfactometry. To identify the volatile compounds emitted from the mummified bodies, thermal desorption tubes filled with Tenax® TA were used as active samplers with calibrated air pumps, collecting 10 L volume with the flow at 100 ml/min. In the case of the display area, the closed display cases during sampling acted as an almost-sealed sampling chamber allowing the accumulation of volatiles. In the storage area this set up was not possible, and the headspace around the mummified bodies was sampled. The environmental parameters of both spaces were determined with HOBO data logger (MX2300 Series, Onset Computer Corp., Pocasset MA) over a 3-h period, measuring on average 27.5 °C and RH 62.1% in the storage area, and 28.1 °C and RH 57.4% in the display area. In both locations, all air samples were collected in four replicates and to verify the reproducibility of the analyses two of them were analyzed at the University of Ljubljana and the other two at the Krakow University of Economics.

At UL, preconditioned thermal desorption tubes (Gerstel, Mülheim an der Ruhr, Germany, 60/80 mesh, 160 mg, metal tube) with Universal Air Sampling Pumps 224-44MTX (SKC, Blandford Forum, UK) were used to collect volatiles. After thermal desorption conducted with a thermal desorption unit (TDSA, Gerstel) with a PTV inlet (CIS, Gerstel) the GC-MS-O analyses of the tubes were performed with gas chromatography-mass spectrometry (GC 7890A, Agilent, Santa Clara CA; MS 5975C, Agilent Technologies) coupled with an olfactory detector port (Phaser pro, GL Sciences, Eindhoven, The Netherlands). The sorbent tubes were thermally desorbed (TD: 20 °C, 1 min; 300 °C, 100 °C/min, 1 min; CIS: −25 °C, 0.5 min; TDS: 240 °C, 10 °C/s, 1 min) in splitless mode and the trapped analytes were injected into the GC-MS-O system. Separation was performed using a VOCOL column (60 m × 0.32 mm × 1.8 µm) (Supelco Inc., Bellefonte PA) and He as the carrier gas at 2.0 ml/min. The instrumental conditions were solvent vent injection, temperature program (40 °C, 2 min; 220 °C, 10 °C/min; 230 °C, 20 °C/min, 10 min); ion source temperature 230 °C; MS transfer temperature 230 °C. MS was operated in TIC mode in the range m/z 43–550 and the compounds were identified based on comparison with the National Institute of Standards and Technology NIST library Mass spectra Library V.2.0. Olfactory port temperature was set constant at 250 °C with synthetic air added to the compounds eluted from the transfer line. Two trained assessors sniffed for 20-minute sessions at room temperature and in an environment isolated of distractions. For each perceived smell they gave a description of the odor and an intensity value from "weak" to "strong". The acquisition of the odors was performed through voice recorder system followed by records transcript, or through odor palette. Only odors detected at least twice out of the four parallels were considered, and descriptors were combined for each smell and compared to descriptors in literature[21,22,24].

At the Krakow University of Economics, pre-conditioned stainless-steel tubes filled with Tenax® TA (35/60 Mesh, Markes, Bridgend, UK) were used for air sampling connected with calibrated pumps (ACTI-VOC PLUS™, Markes). The desorption of analytes was conducted with a TD unit (Autosampler ULTRA-xr, Markes; TD: UNITY-xr, Markes) with pre purge for trap 40 mL/min, 1.0 min; tube desorption 230 °C, 3.5 min with flow at 40 mL/min with trap online splitless; trapping temperature -30 °C; trap heating rate 250 °C, 5.0 min. After the injection, separation and detection were done with a GC-MS-O (GC: TRACE 1610, Thermo Fisher, Waltham MA; MS: ISQ 7610, Thermo Fisher). The analysis was performed with a VOCOL® column (60 m x 0.25 mm x 1.5 µm; Merck) and the chromatographic parameters were the same used at UL. The mass range explored was 33-660 m/z and the compounds were identified based on the mass spectral NIST20 library. The olfactory port used and the parameters chosen for the olfactory detection were the same as those used at UL.

The pest oil mixture, as produced and used by museum staff for disinfection consists of clove oil, camphor oil, peppermint extract, basil oil, lemon oil, orange oil, and cinnamon oil. A sample was collected for sensory and GC-MS-O analysis.

4.3 Microbiological analysis

Analysis of microbial air quality with the sedimentation method. Microbial air quality analysis was conducted using a passive sedimentation method. Plate Count Agar (PCA) and Potato Dextrose Agar (PDA, both purchased in already prepared sterile plastic Petri dishes, Oxoid, Basingstoke, UK) were used to isolate bacteria and fungi,

respectively. At the sampling location the lid of Petri dish was removed exposing the medium to microorganisms in the air for 10 min. Subsequently, the Petri dish were covered and sealed with parafilm, and then cultivated in the laboratory in incubators at 28 ± 2 °C. Bacterial colony growth was observed after 24, 48, and 72 h, while mold colony observation on PDA media extended from 24 h to 7 days. Colonies were enumerated, and the quantity of bacteria and fungi in the air at each sampling site was calculated and expressed as Colony Forming Units per cubic meter (CFU/m^3). Then, microorganisms were isolated to the pure culture and identified using the MALDI-TOF apparatus (MALDI Biotyper®, Bruker, Billerica MA).

Microbial analysis of the mummified bodies. The microbiological contamination of linen wrappings was assessed with the swabbing method. Before sampling, each mummified body was visually inspected to check visible changes that could be caused by active microorganisms. Sampling was performed by swabbing (Amies, Canelli AT, Italy) on a 25 cm^2 area. The material collected was then inoculated in the laboratory on the PCA and PDA media in Petri dishes, in incubators at 28 ± 2 °C. Bacterial colony growth was observed after 24, 48, and 72 h, while mold colony observation on PDA media extended from 24 h to 7 days. Subsequently, microorganisms were isolated to the pure culture and identified using the MALDI-TOF Biotyper®.

4.4 Data analysis

OriginPro® 2024 was used for hierarchical cluster analysis, based on cluster averages and squared Euclidean distance, with and without variable standardization.

5. Microbiological analysis: results

The microorganisms sampled on microbial media from the museum atmosphere were incubated and then isolated and identified using MALDI-TOF. Supplementary Tables 4 and 5, show that the microbial quality of the air varies significantly. The concentration of bacteria is in the range from 240 to 2516 CFU/m^3 of air, whereas the measured concentration of molds varied from 78 to 1258 CFU/m^3 (Supplementary Table 4). The bacterial concentration in the analyzed air inside the museum is within acceptable levels and typical for such interiors, especially considering the regional climate conditions[25]. However, at two sampling locations, one in the storage area (next to M3) and above the display case containing M6-M7-M8, the bacterial concentration was noticeably higher than at other locations. For fungi, it is noticeable that at sampling point next to M4, the mold concentration was significantly higher than at other locations, but still within acceptable limits, consistent with measurements from other museums[25]. At no sampling point were elevated concentrations of both bacteria and fungi detected simultaneously.

The data presented in Supplementary Table 5 indicates that the dominant bacteria are *Bacillus sp.* species. These bacteria with proteolytic and cellulolytic properties are common in the environment including museum environments[26]. Two of the identified species, *Brachybacterium nesterenkovii* and *Paenibacillus lactis,* are typically found in milk products, and are likely to indicate potential contamination by museum staff rather than the original use of milk products for the mummification. The dominant fungi include *Rhizopus oryzae, Penicillium chrysogenum, Aspergillus flavus,* and *Aspergillus niger.* These species are also common environmental species isolated from the air of many museums[25-27]. Their proteolytic and cellulolytic properties[28, 29] can contribute to the biodeterioration of various materials containing natural polymers.

Supplementary Table 4. The concentration of bacteria and molds in the air. Numbers (1-9) correspond to the location of the mummified bodies (M1-M9).

Sampling location	Concentration of bacteria in the air (CFU/m^3)	Concentration of mold in the air (CFU/m^3)
	**Storage**	
**General area storage**	550 ± 51	236 ± 34
**1**	314 ± 45	78 ± 43
**2**	1258 ± 56	865 ± 54
**3**	2438 ± 72	629 ± 43
**4**	393 ± 43	1258 ± 35
**5**	236 ± 32	551 ± 84
	**Display**	

**6**	240 ± 21	547 ± 73
**7**	865 ± 34	157 ± 62
**8**	708 ± 43	550 ± 23
**Above the display case (M6-M7-M8)**	-	543 ± 88
**Above the display case (M6-M7-M8)**	2516 ± 68	-
**Next to the display case (M6-M7-M8)**	314 ± 23	629 ± 46
**9**	865 ± 32	236 ± 32

Supplementary Table 5. Microorganisms isolated from the air using petri dishes, sampled in the various locations in the museum. Numbers (1-9) correspond to the locations of the mummified bodies (M1-M9).

Sampling location	Bacteria	Molds
1	Bacillus cereus	Rhizopus oryzae
2	Bacillus velezensis,	Aspergillus flavus, Penicillium chrysogenum, Rhizopus oryzae
3	Brachybacterium nesterenkovii, Paenibacillus lactis, Bacillus subtilis, Bacillus atrophaeus, Bacillus pumilus, Pseudomonas stutzeri,	Aspergillus flavus
4	Kocuria palustris, Bacillus altitudinis, Bacillus pumilus, Priestia endophytica	Aspergillus niger
5	Bacillus pumilus,	Cladosporium cladosporioides
6	Bacillus velezensis,	Rhizopus oryzae
7	Bacillus subtilis	Penicillium chrysogenum, Aspergillus flavus
8	Bacillus velezensis, Bacillus pseudomycoides	Aspergillus flavus
9	Bacillus pumilus, Bacillus subtilis	Aspergillus niger

Supplementary Table 6. Microorganisms isolated from the swabs at the selected sampling locations. Numbers (1-9) correspond to the location of the mummified bodies (M1-M9).

Sampling location	Bacteria	Molds
M1	*Bacillus subtilis*	*Aspergillus parasiticus, Aspergillus niger*
M2	*Priestia megaterium, Cytobacillus oceanisediminis*	*Rhizopus oryzae, Aspergillus flavus*
M3	*Bacillus subtilis, Bacillus pumilus*	*Aspergillus terreus*
M4	*Bacillus pumilus*	*Aspergillus flavus*
M5	*Bacillus subtilis*	*Cladosporium cladosporioides, Aspergillus niger, Cladosporium herbarum*
M6	*Bacillus subtilis, Bacillus pumilus*	*Penicillum chrysogenum*
M7	*Bacillus subtilis, Bacillus pumilus*	*Aspergillus niger,*
M8	*Bacillus subtilis*	*Penicillium oxalicum, Rhizopus delemar*
M9	*Bacillus subtilis, Kocuria rosea*	*Candida tropicalis, Pseudomonas rhizosphaerae, Cladosporium cladosporioides, Talaromyces sp.*

6. GC-MS-O chromatograms/olfactograms

In the following, the MS chromatograms/olfactograms are presented for the individual analyses of Tenax® tubes.

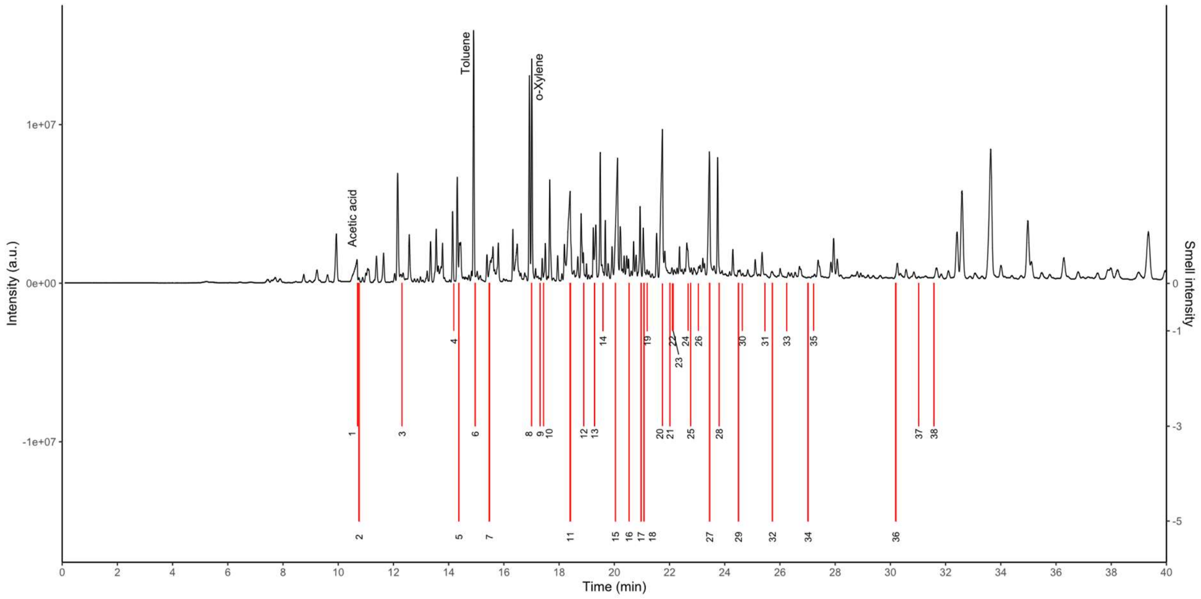

Supplementary Figure 1. MS chromatogram (black) and olfactogram (red) for the sample M1 (subsample 1). The numeric labels refer to SI3.

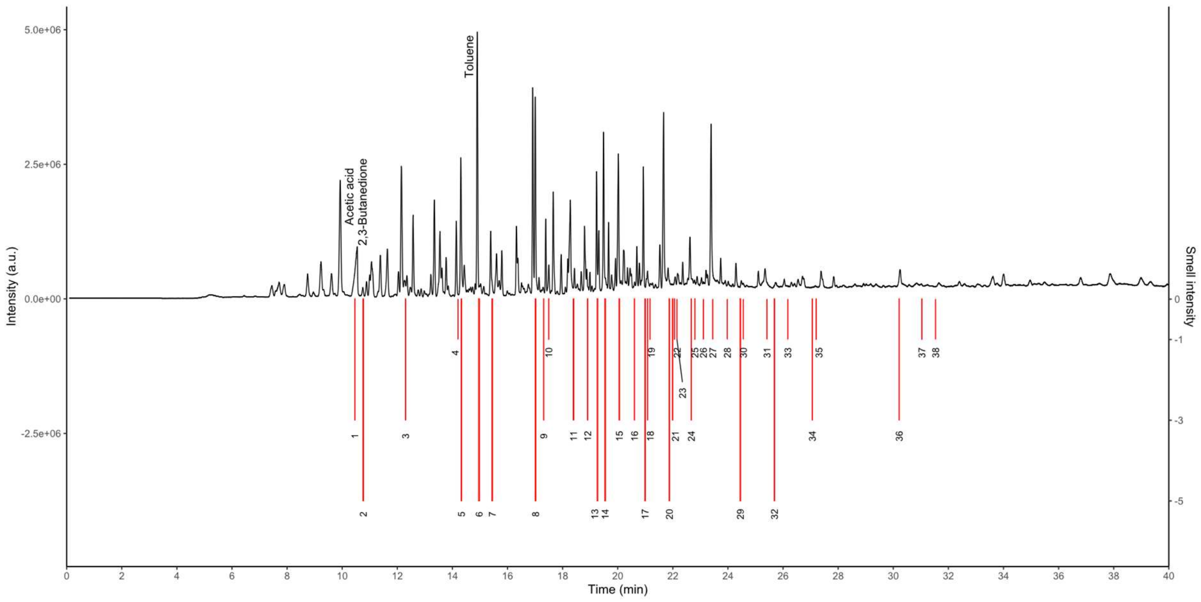

Supplementary Figure 2. MS chromatogram (black) and olfactogram (red) for the sample M1 (subsample 2). The numeric labels refer to SI3.

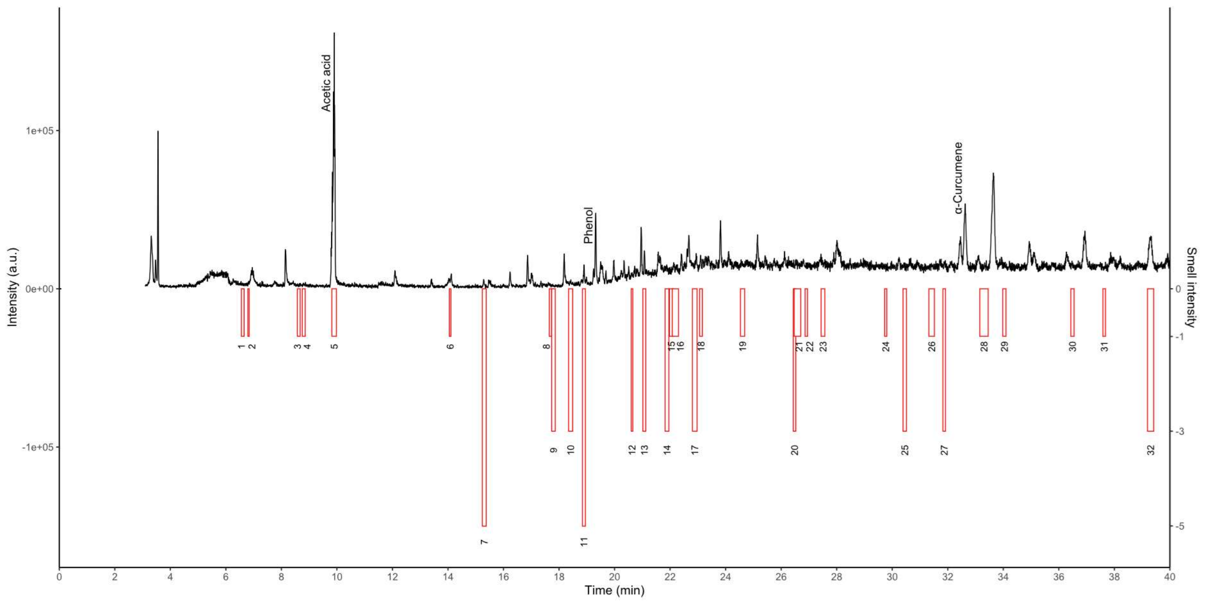

Supplementary Figure 3. MS chromatogram (black) and olfactogram (red) for the sample M1 (subsample 3). The numeric labels refer to SI3.

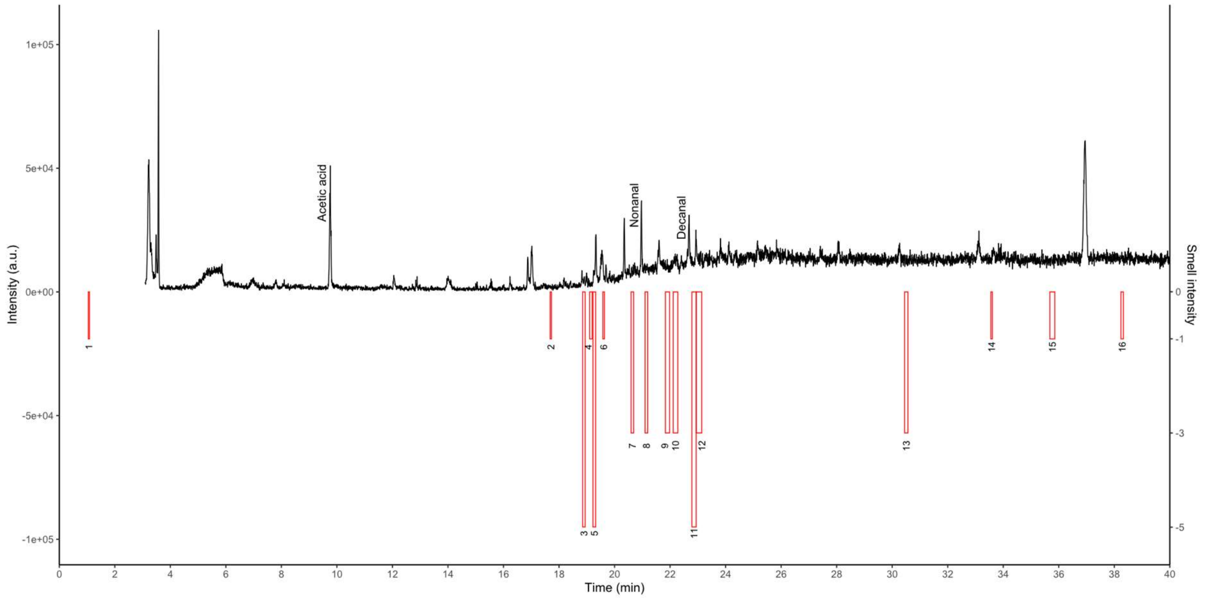

Supplementary Figure 4. MS chromatogram (black) and olfactogram (red) for the sample M1 (subsample 4). The numeric labels refer to SI3.

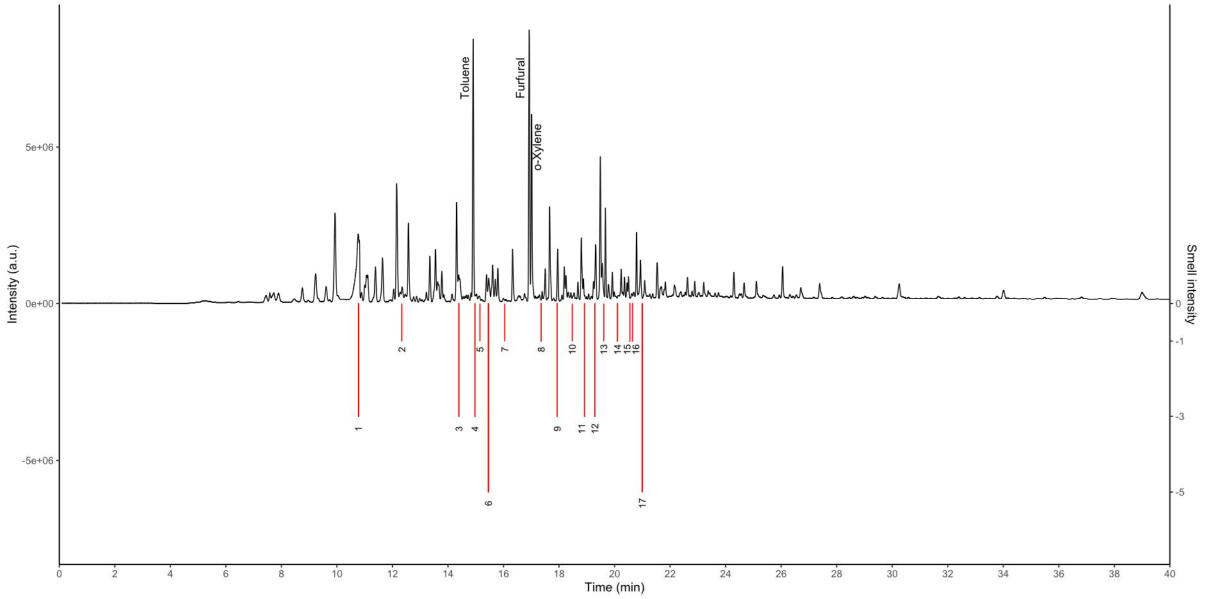

Supplementary Figure 5. MS chromatogram (black) and olfactogram (red) for the sample M2 (subsample 1). The numeric labels refer to SI3.

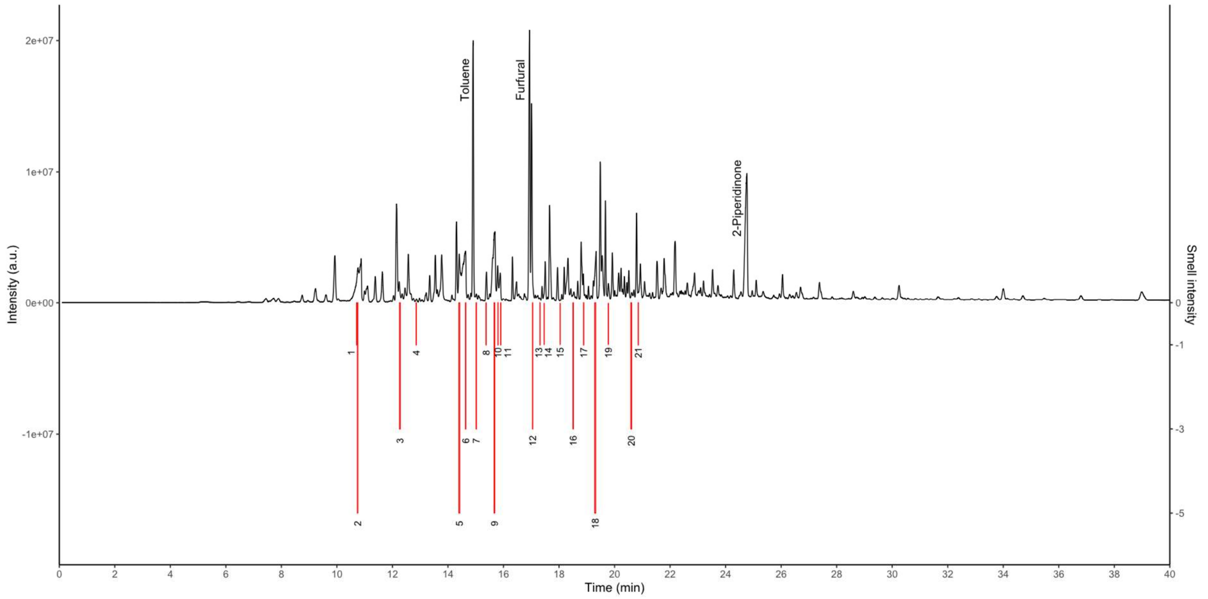

Supplementary Figure 6. MS chromatogram (black) and olfactogram (red) for the sample M2 (subsample 2). The numeric labels refer to SI3.

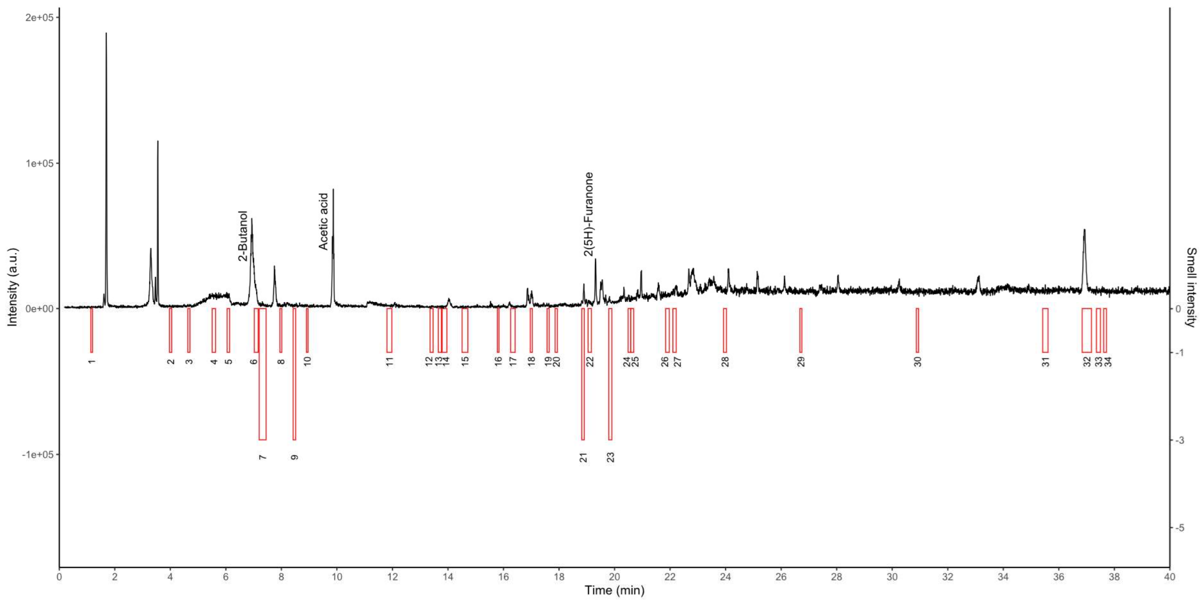

Supplementary Figure 7. MS chromatogram (black) and olfactogram (red) for the sample M2 (subsample 3). The numeric labels refer to SI3.

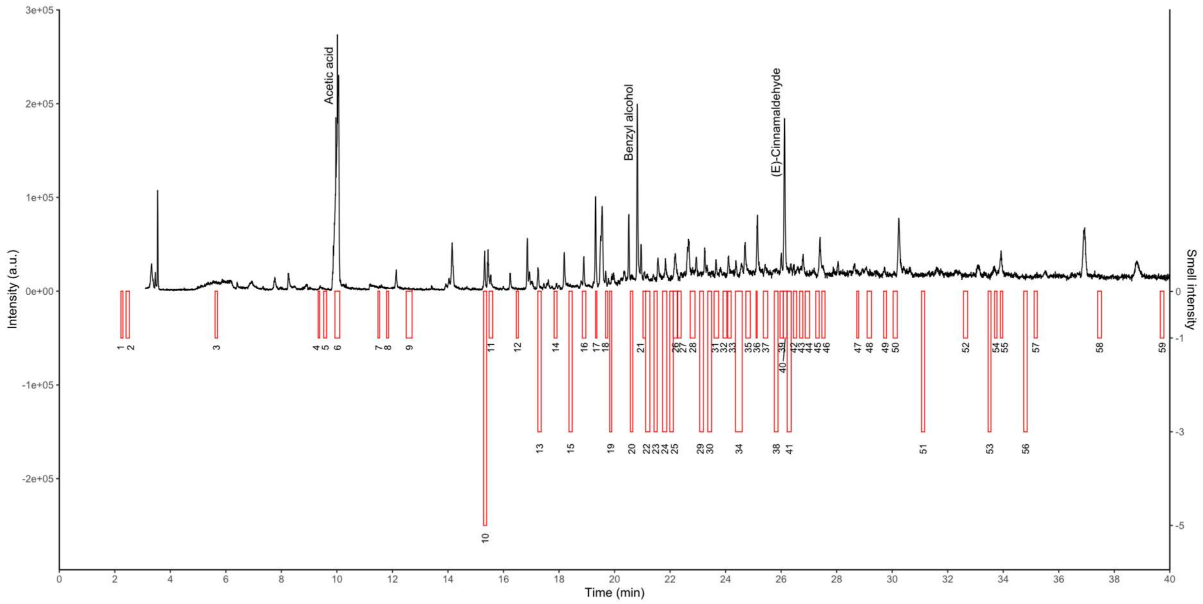

Supplementary Figure 8. MS chromatogram (black) and olfactogram (red) for the sample M2 (subsample 4). The numeric labels refer to SI3.

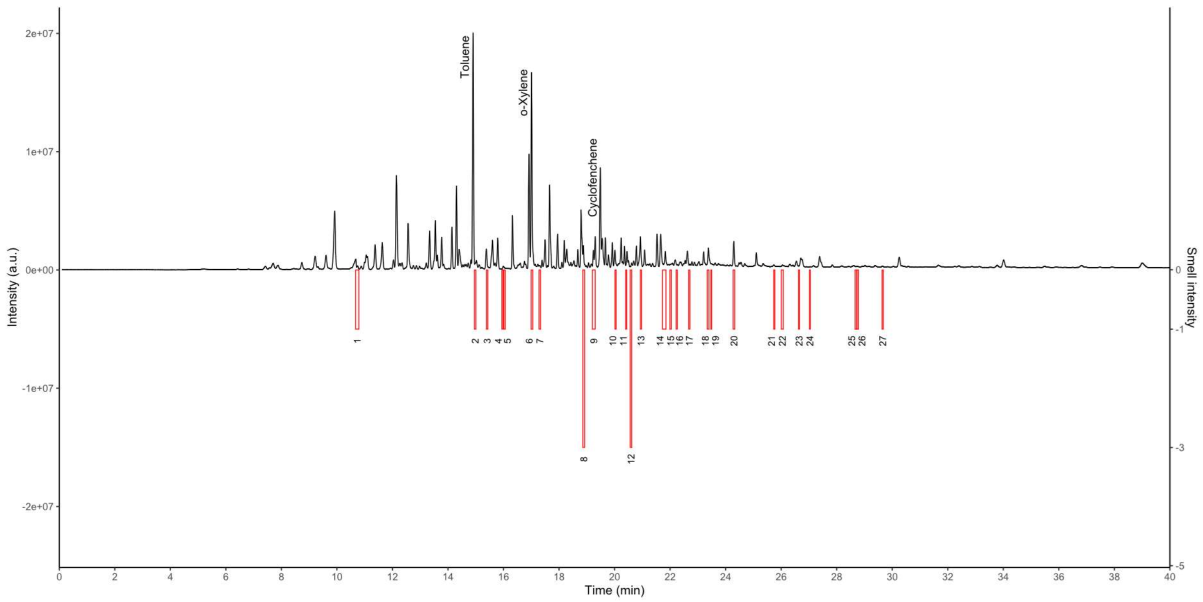

Supplementary Figure 9. MS chromatogram (black) and olfactogram (red) for the sample M3 (subsample 1). The numeric labels refer to SI3.

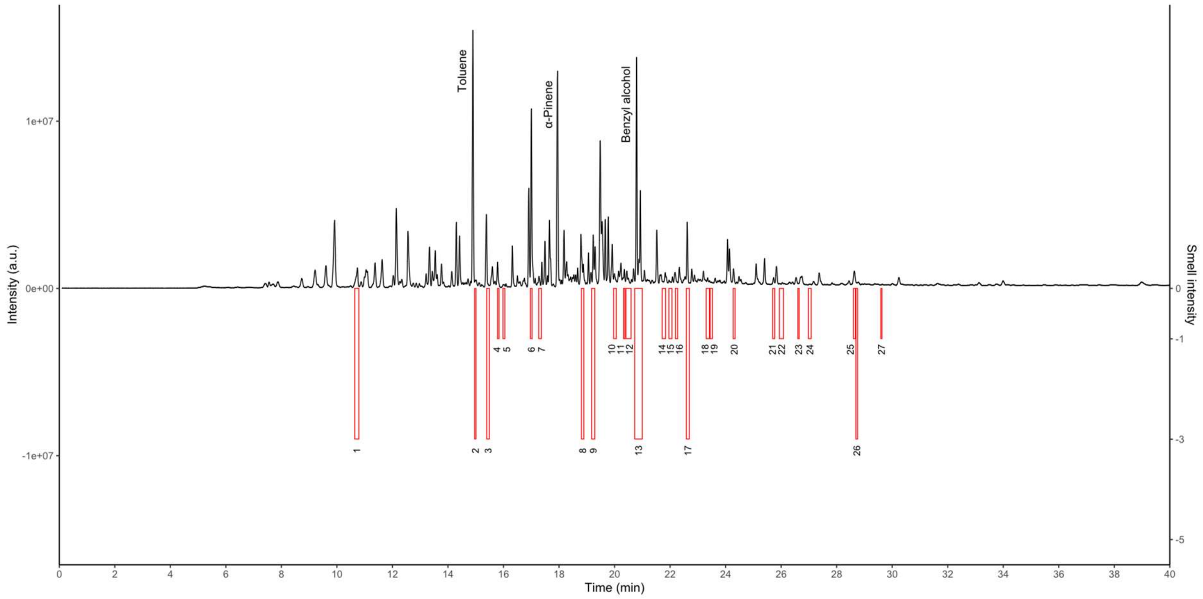

Supplementary Figure 10. MS chromatogram (black) and olfactogram (red) for the sample M3 (subsample 2). The numeric labels refer to SI3.

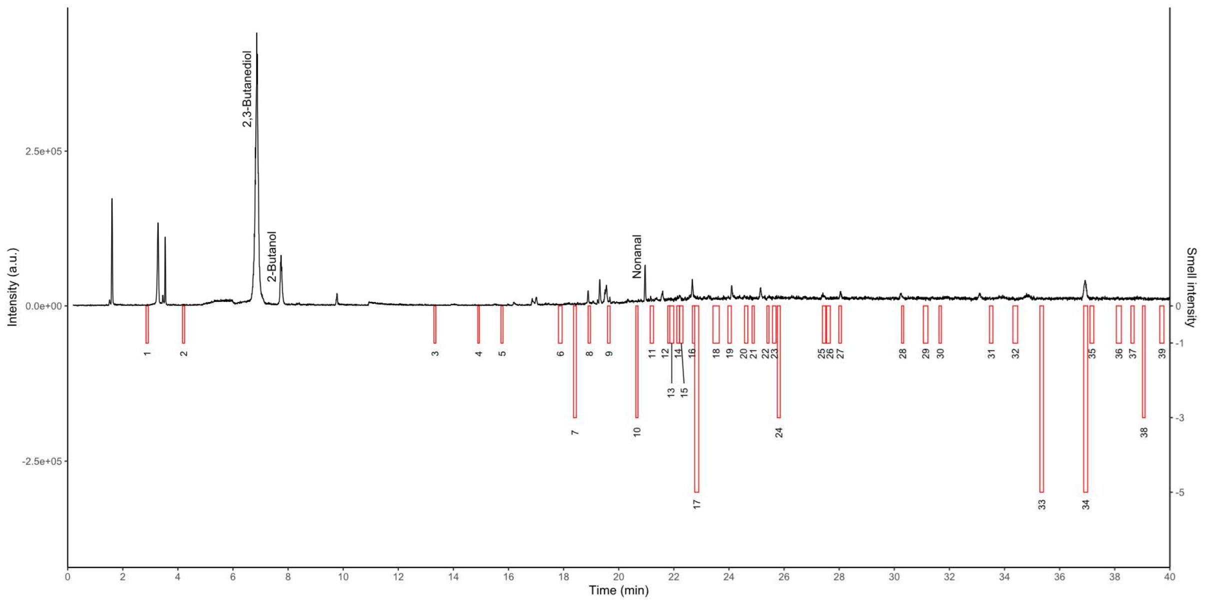

Supplementary Figure 11. MS chromatogram (black) and olfactogram (red) for the sample M3 (subsample 3). The numeric labels refer to SI3.

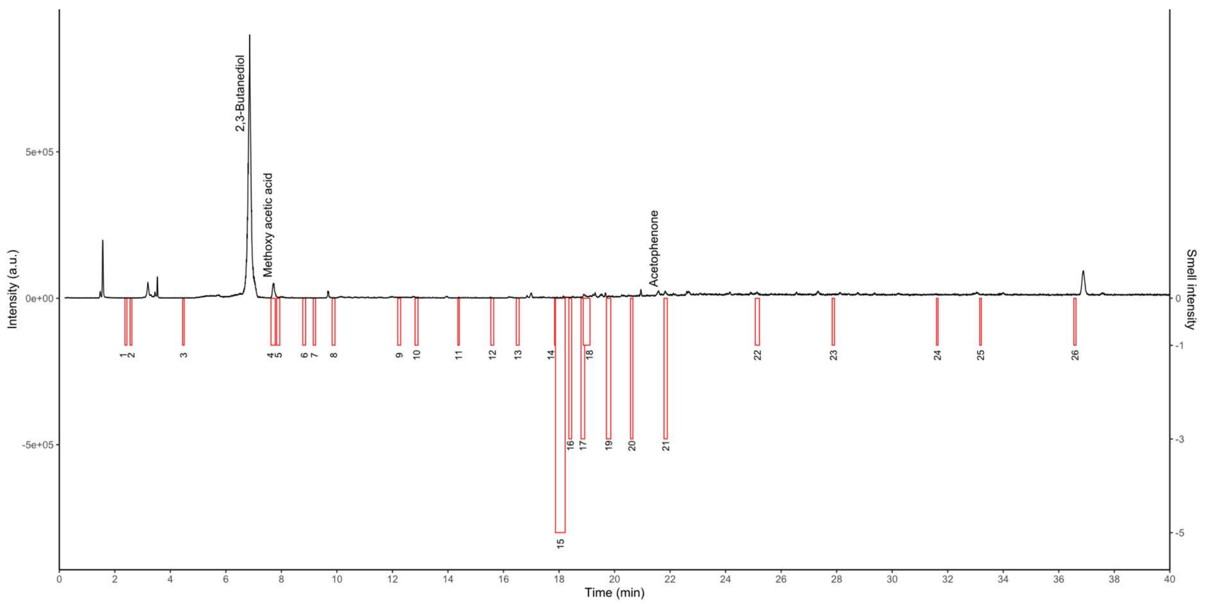

Supplementary Figure 12. MS chromatogram (black) and olfactogram (red) for the sample M3 (subsample 4). The numeric labels refer to SI3.

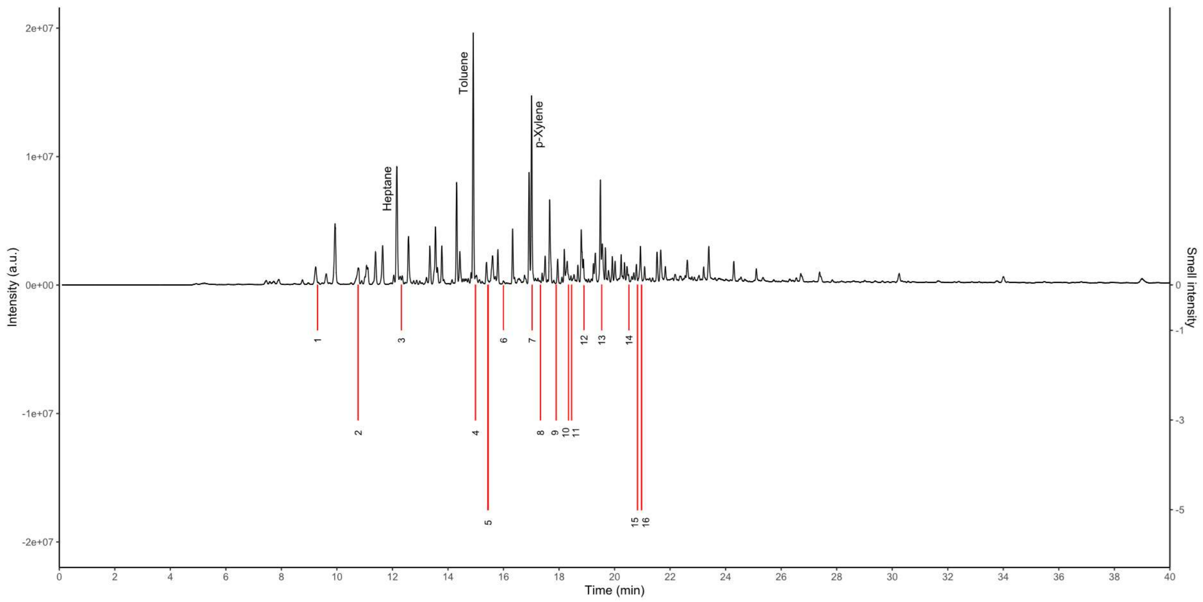

Supplementary Figure 13. MS chromatogram (black) and olfactogram (red) for the sample M4 (subsample 1). The numeric labels refer to SI3.

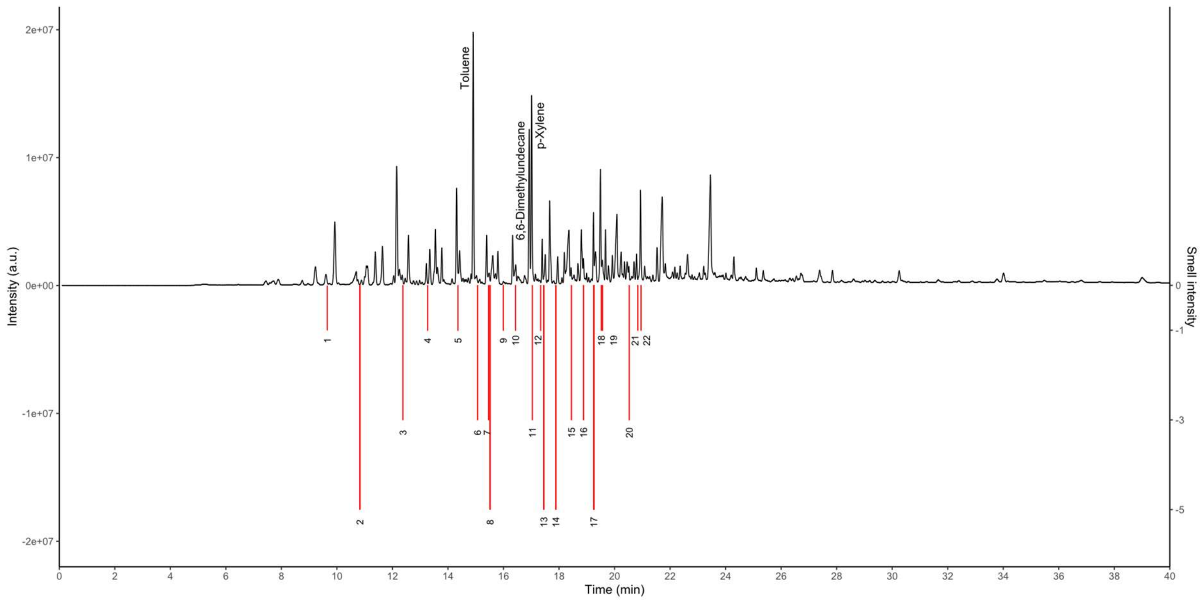

Supplementary Figure 14. MS chromatogram (black) and olfactogram (red) for the sample M4 (subsample 2). The numeric labels refer to SI3.

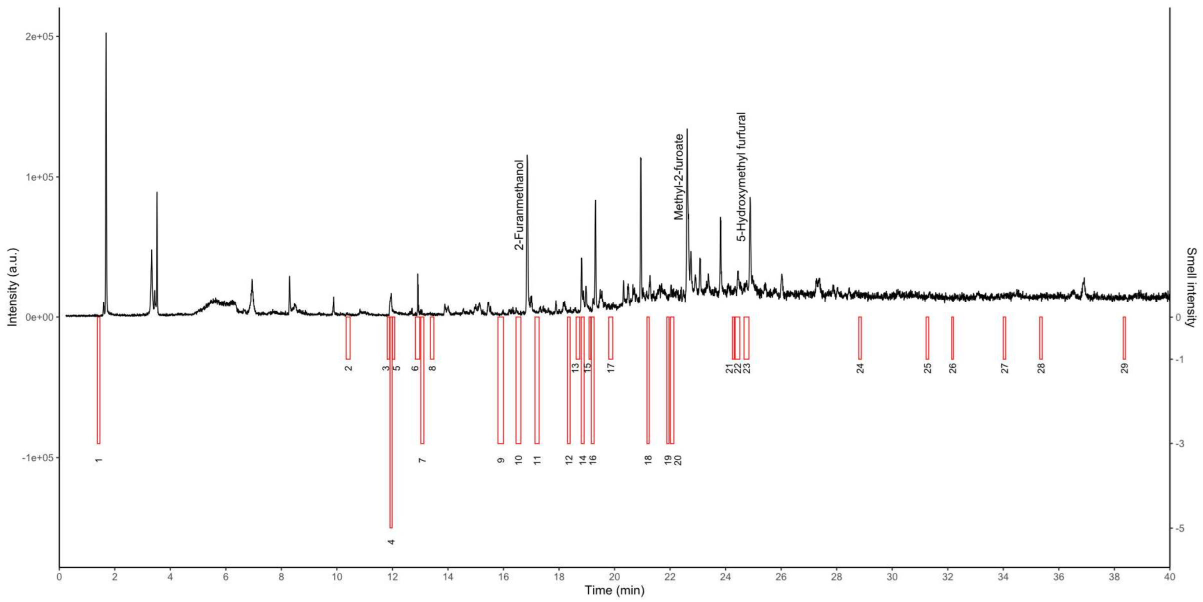

Supplementary Figure 15. MS chromatogram (black) and olfactogram (red) for the sample M4 (subsample 3). The numeric labels refer to SI3.

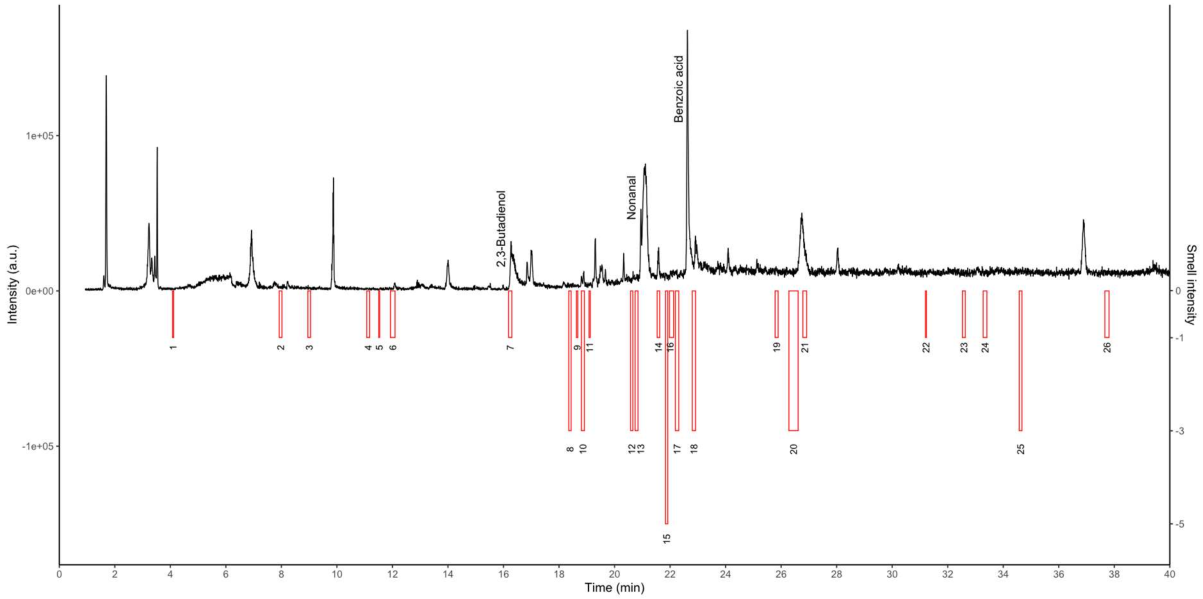

Supplementary Figure 16. MS chromatogram (black) and olfactogram (red) for the sample M4 (subsample 4). The numeric labels refer to SI3.

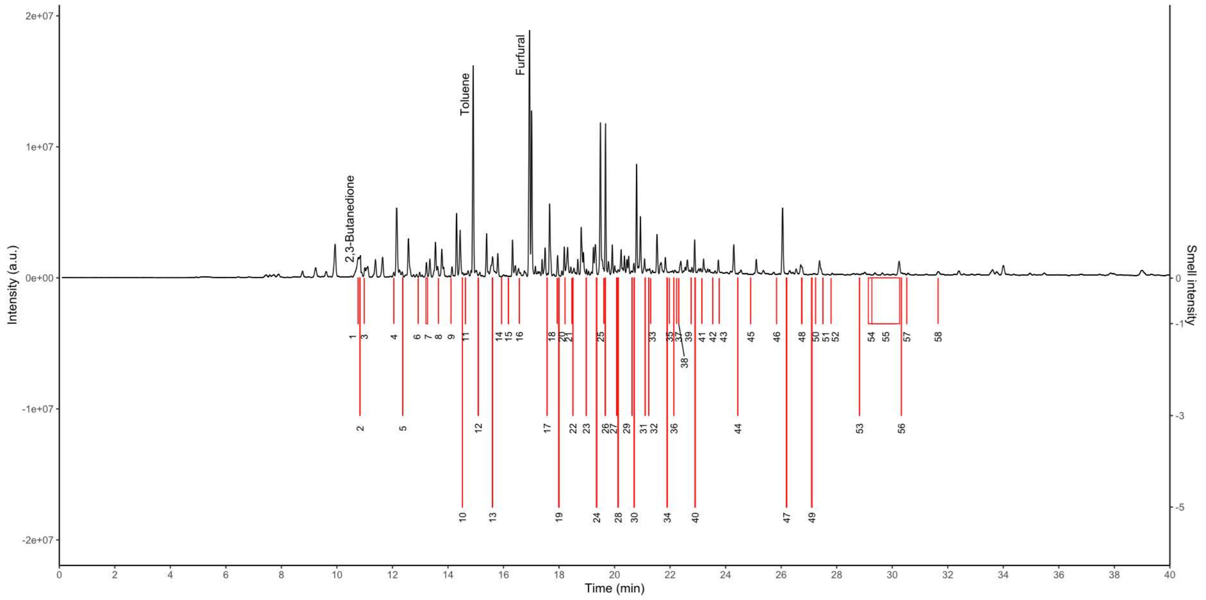

Supplementary Figure 17. MS chromatogram (black) and olfactogram (red) for the sample M5 (subsample 1). The numeric labels refer to SI3.

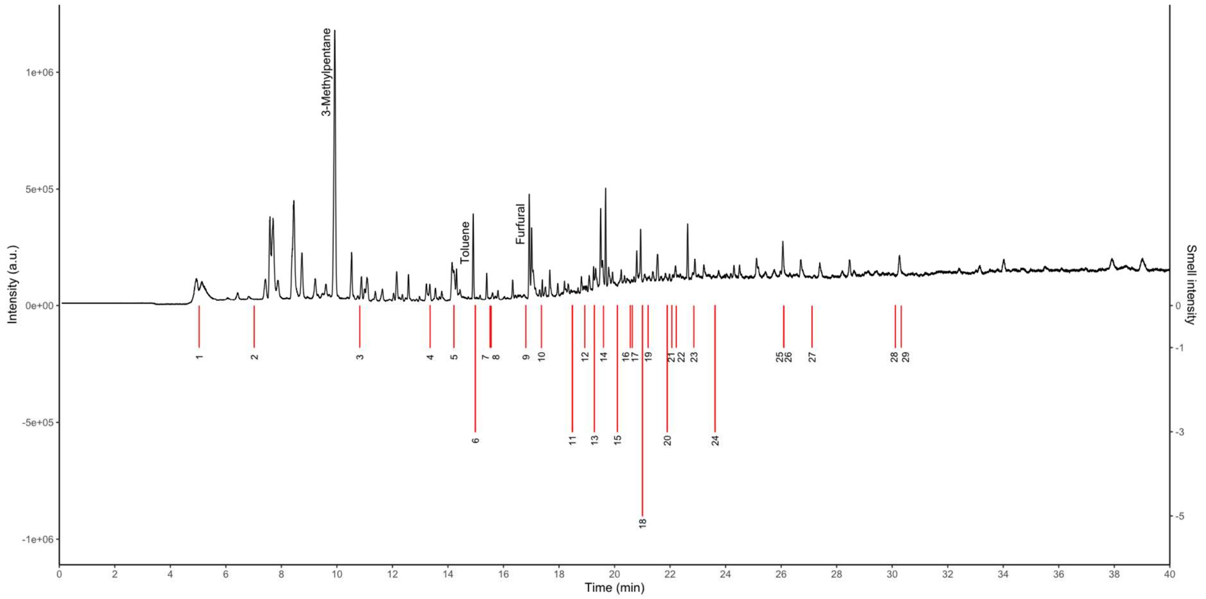

Supplementary Figure 18. MS chromatogram (black) and olfactogram (red) for the sample M5 (subsample 2). The numeric labels refer to SI3.

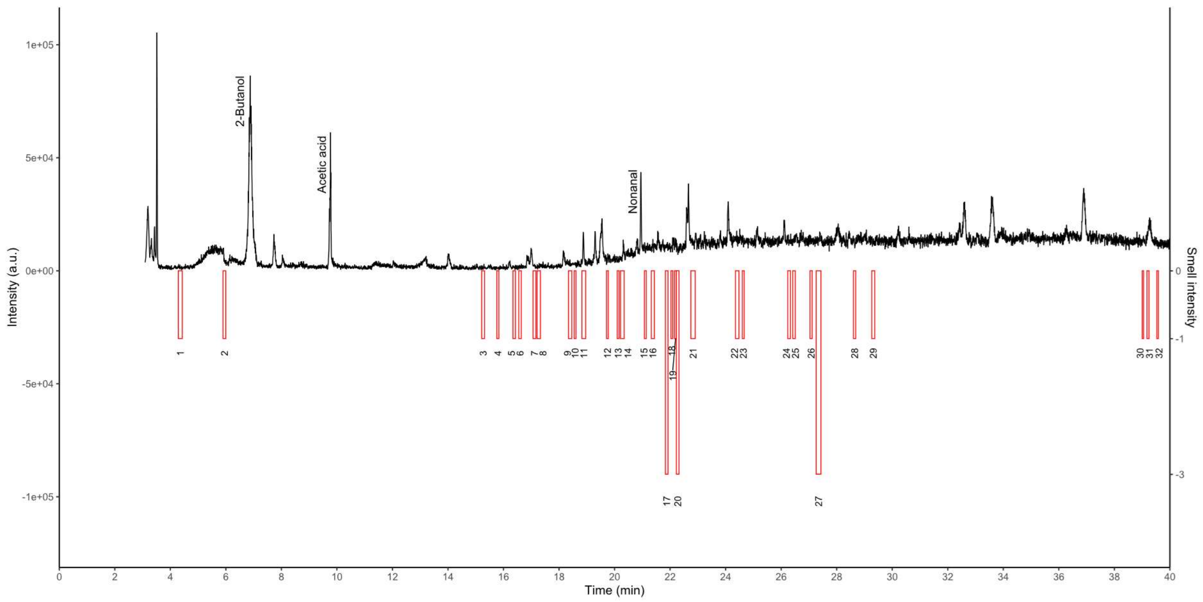

Supplementary Figure 19. MS chromatogram (black) and olfactogram (red) for the sample M5 (subsample 3). The numeric labels refer to SI3.

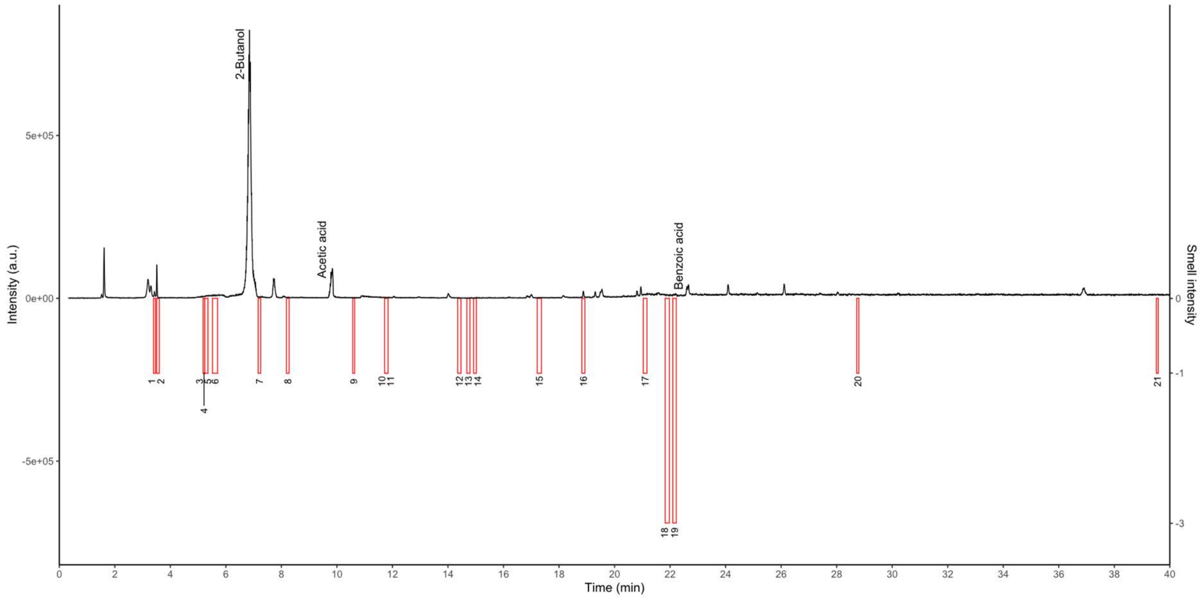

Supplementary Figure 20. MS chromatogram (black) and olfactogram (red) for the sample M5 (subsample 4). The numeric labels refer to SI3.

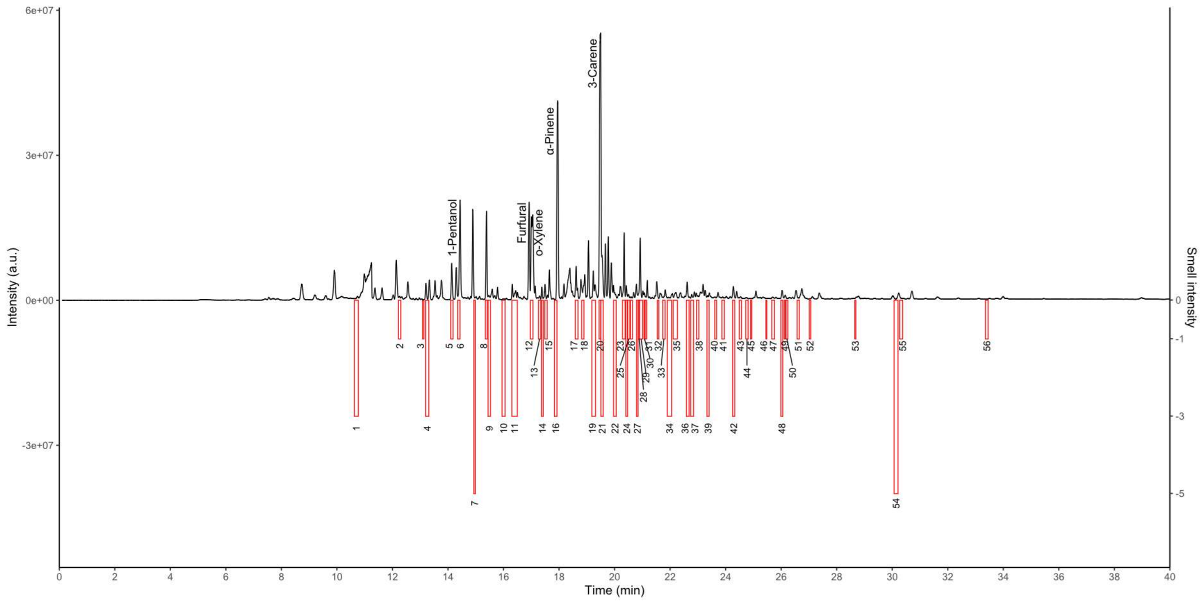

Supplementary Figure 21. MS chromatogram (black) and olfactogram (red) for the sample M6 (subsample 1). The numeric labels refer to SI3.

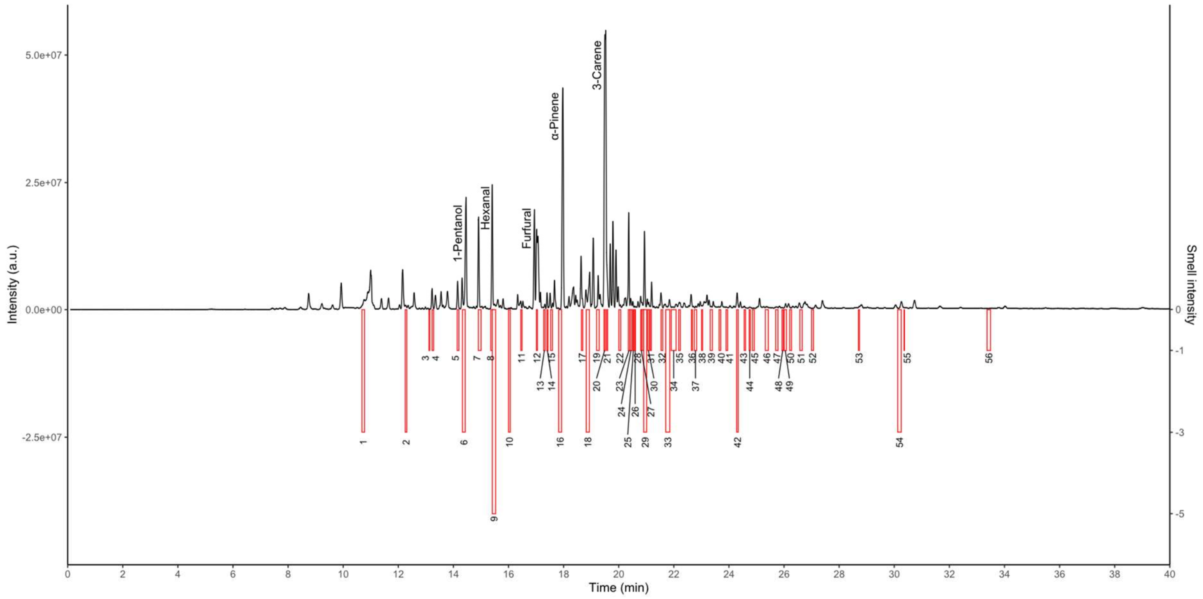

Supplementary Figure 22. MS chromatogram (black) and olfactogram (red) for the sample M6 (subsample 2). The numeric labels refer to SI3.

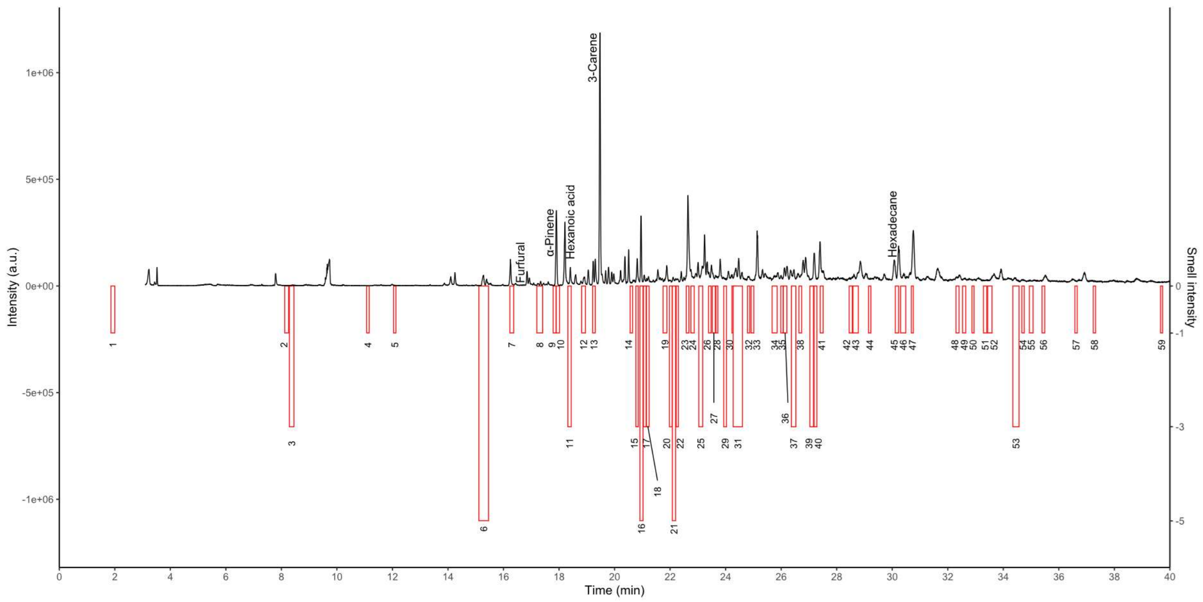

Supplementary Figure 23. MS chromatogram (black) and olfactogram (red) for the sample M6 (subsample 3). The numeric labels refer to SI3.

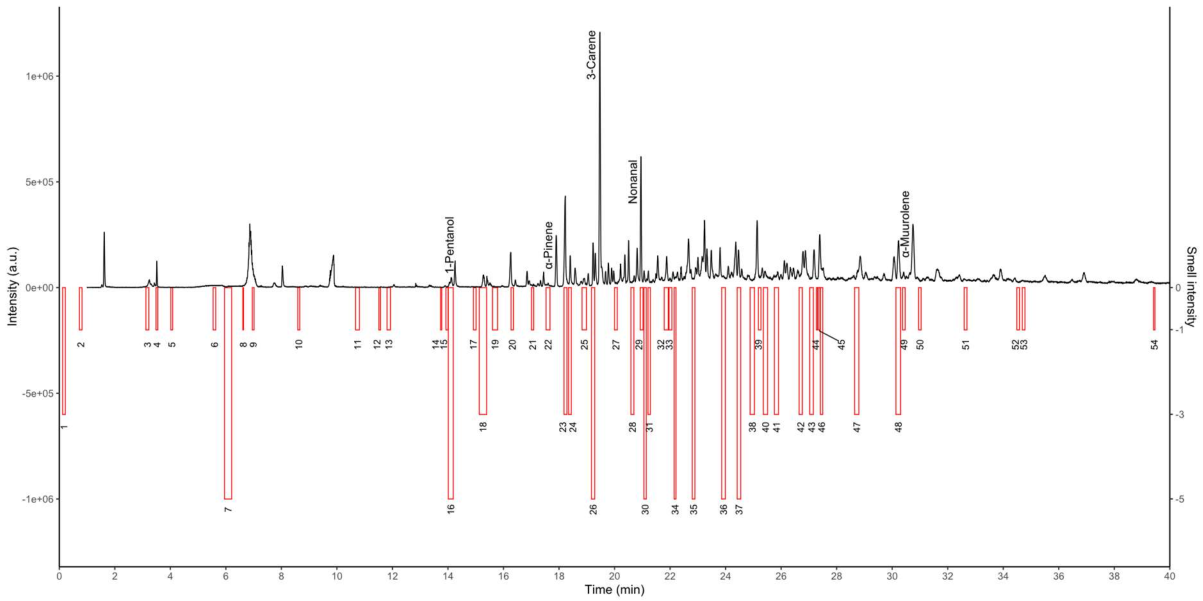

Supplementary Figure 24. MS chromatogram (black) and olfactogram (red) for the sample M6 (subsample 4). The numeric labels refer to SI3.

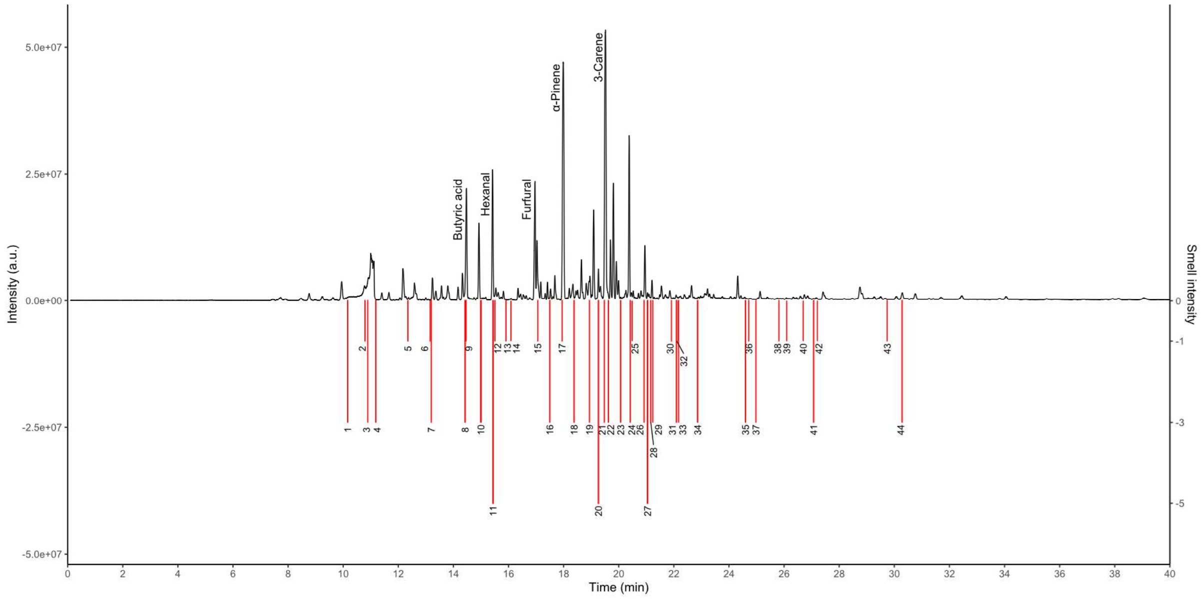

Supplementary Figure 25. MS chromatogram (black) and olfactogram (red) for the sample M7 (subsample 1). The numeric labels refer to SI3.

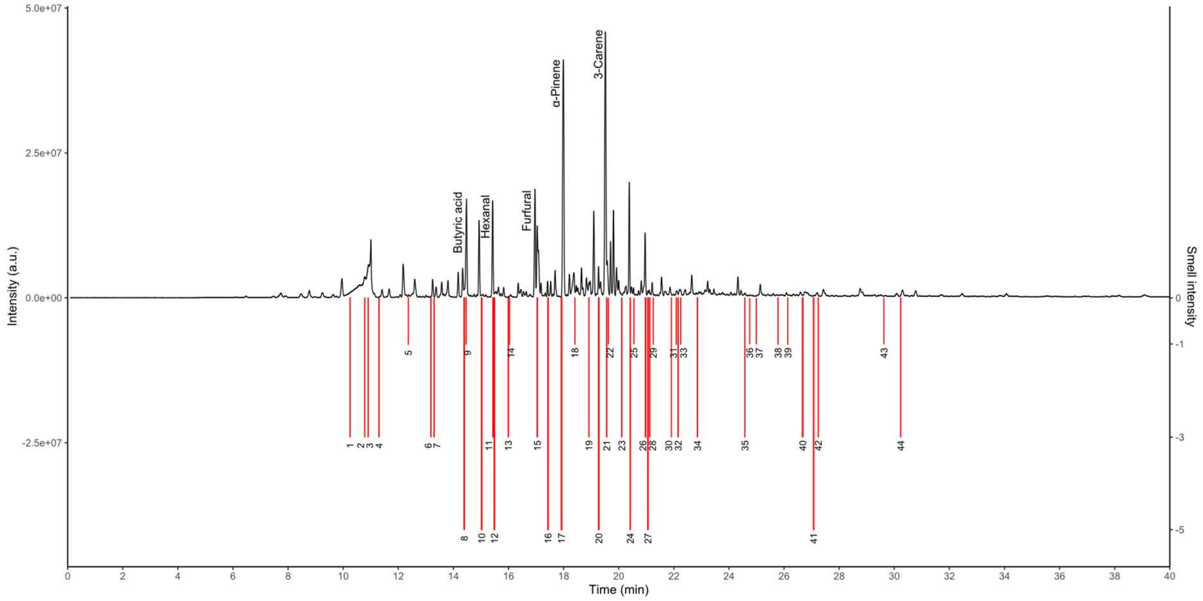

Supplementary Figure 26. MS chromatogram (black) and olfactogram (red) for the sample M7 (subsample 2). The numeric labels refer to SI3.

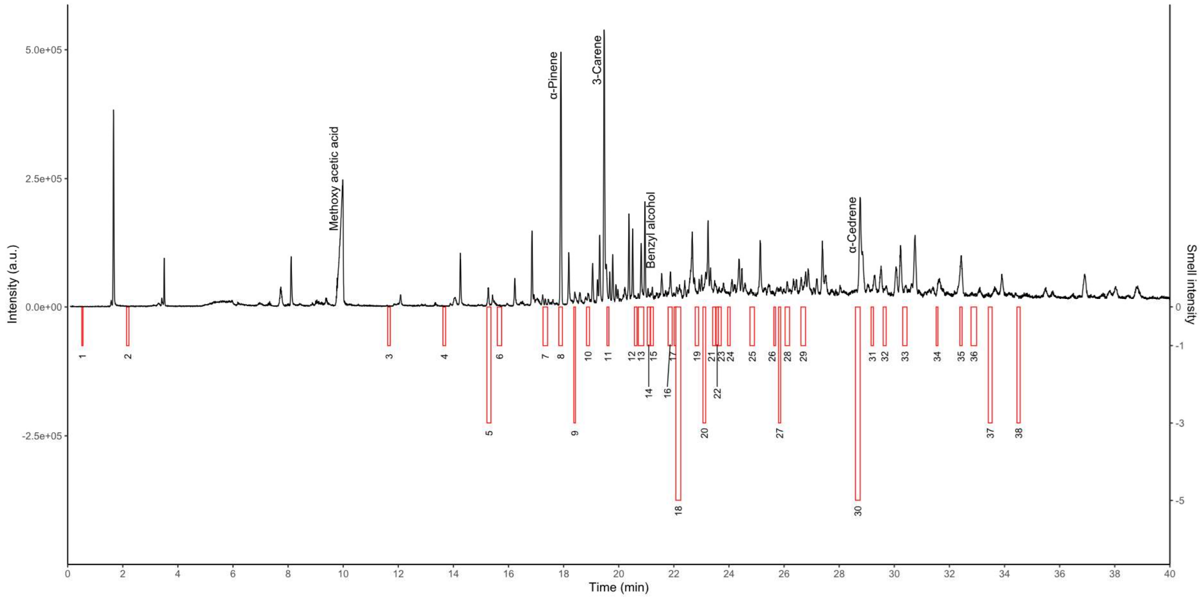

Supplementary Figure 27. MS chromatogram (black) and olfactogram (red) for the sample M7 (subsample 3). The numeric labels refer to SI3.

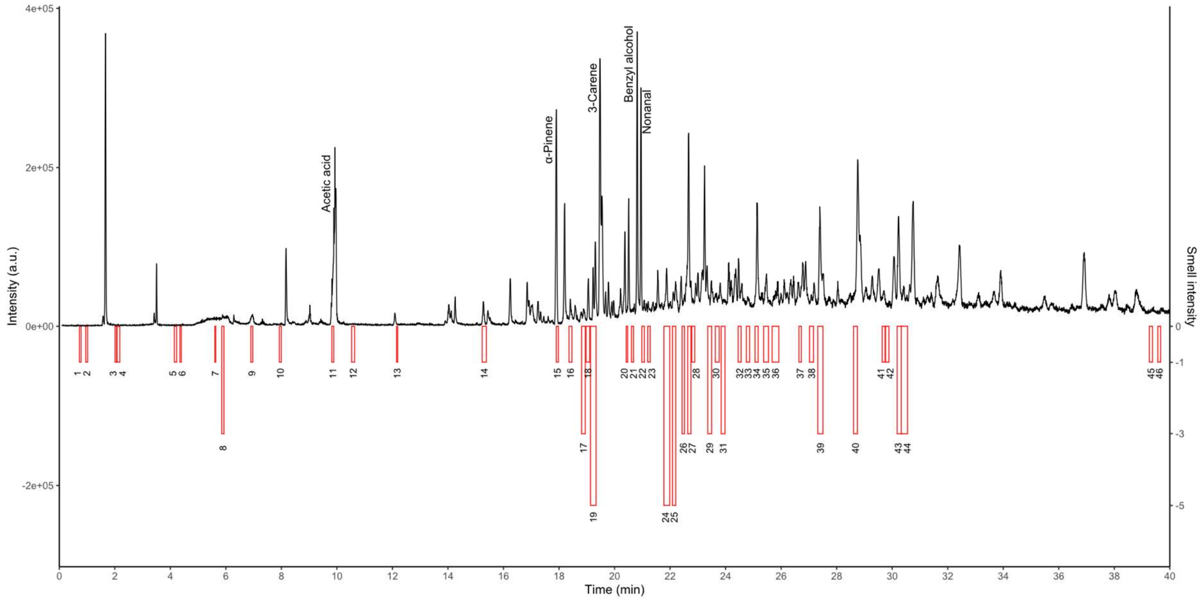

Supplementary Figure 28. MS chromatogram (black) and olfactogram (red) for the sample M7 (subsample 4). The numeric labels refer to SI3.

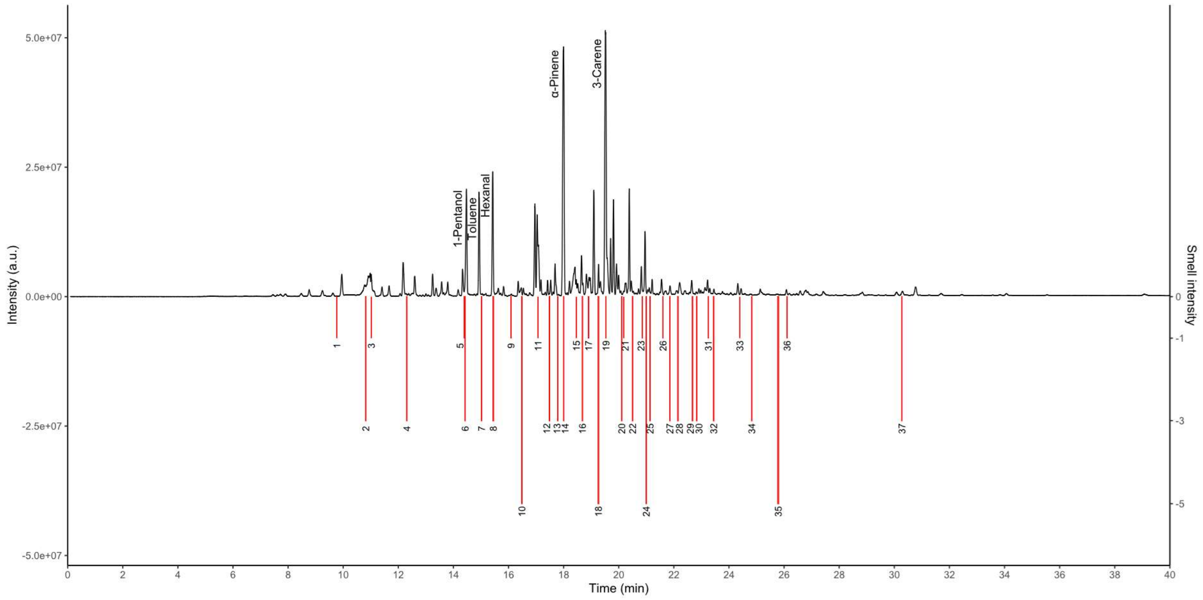

Supplementary Figure 29. MS chromatogram (black) and olfactogram (red) for the sample M8 (subsample 1). The numeric labels refer to SI3.

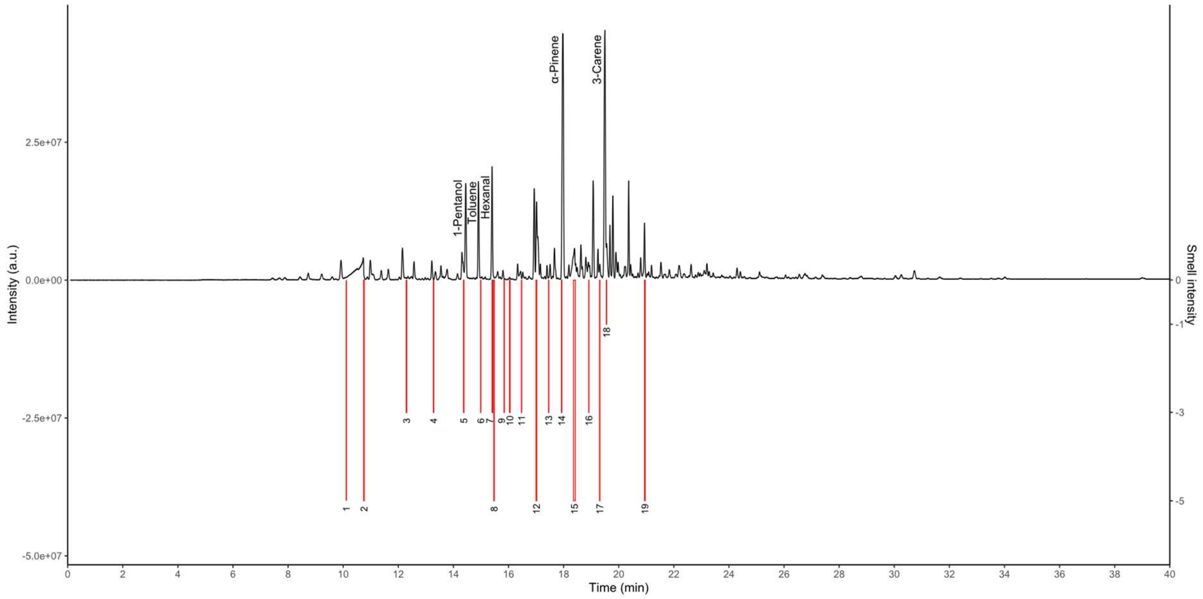

Supplementary Figure 30. MS chromatogram (black) and olfactogram (red) for the sample M8 (subsample 2). The numeric labels refer to SI3.

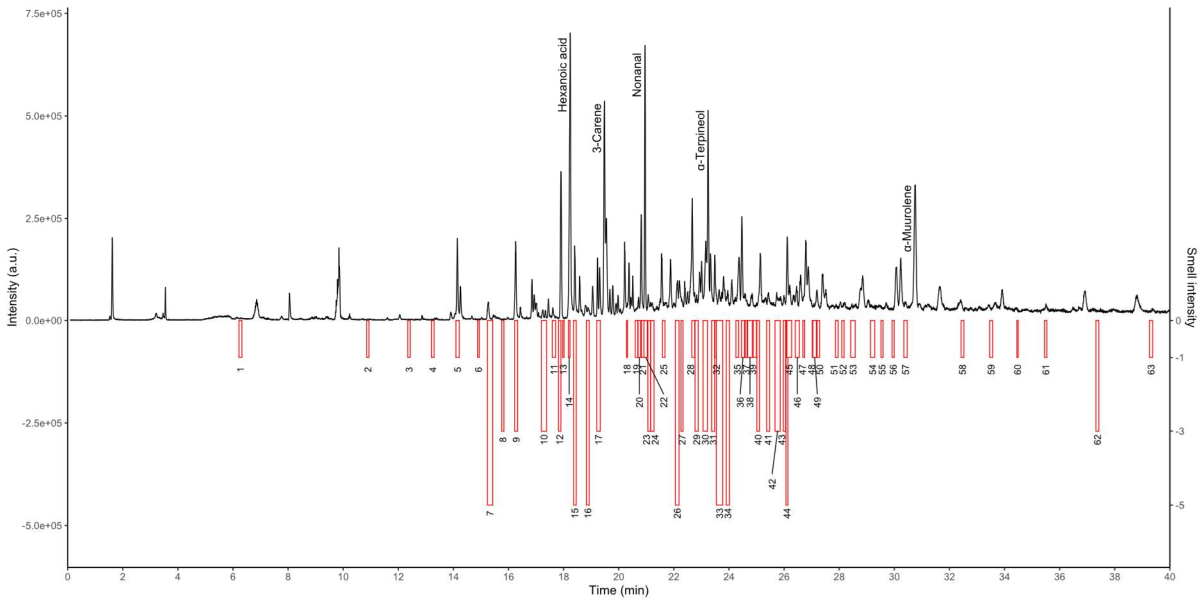

Supplementary Figure 31. MS chromatogram (black) and olfactogram (red) for the sample M8 (subsample 3). The numeric labels refer to SI3.

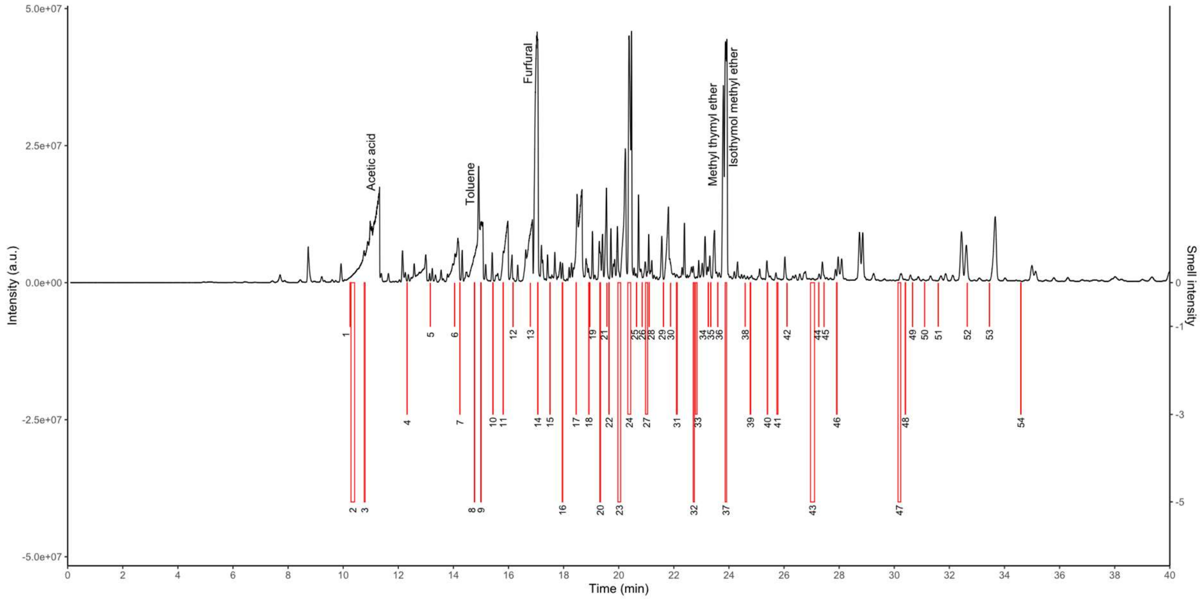

Supplementary Figure 32. MS chromatogram (black) and olfactogram (red) for the sample M9 (subsample 1). The numeric labels refer to SI3.

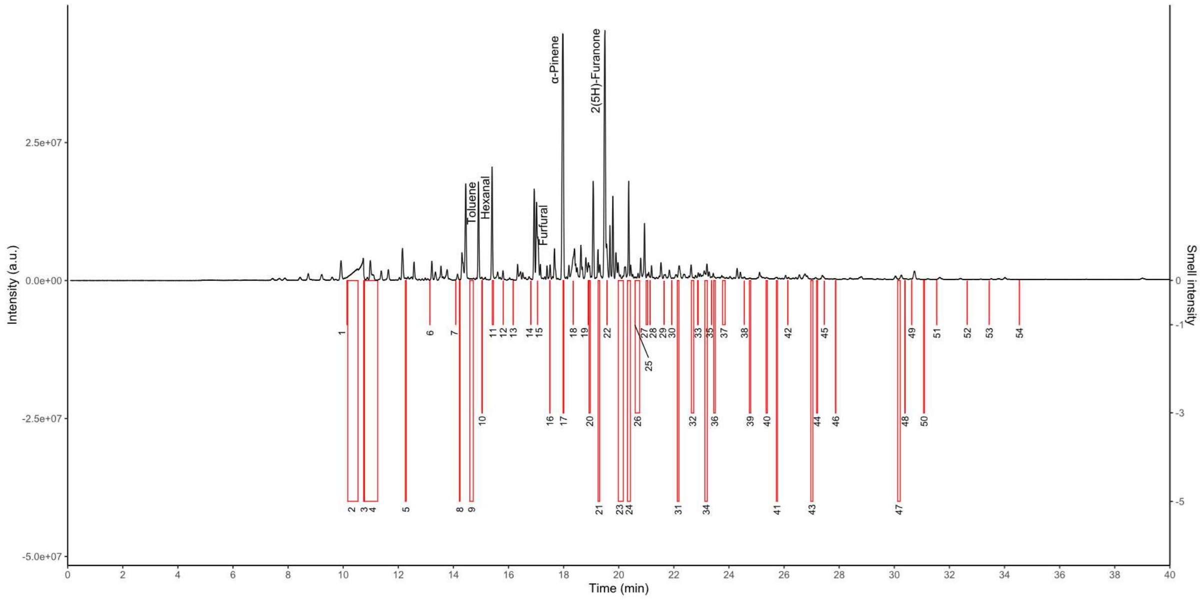

Supplementary Figure 33. MS chromatogram (black) and olfactogram (red) for the sample M9 (subsample 2). The numeric labels refer to SI3.

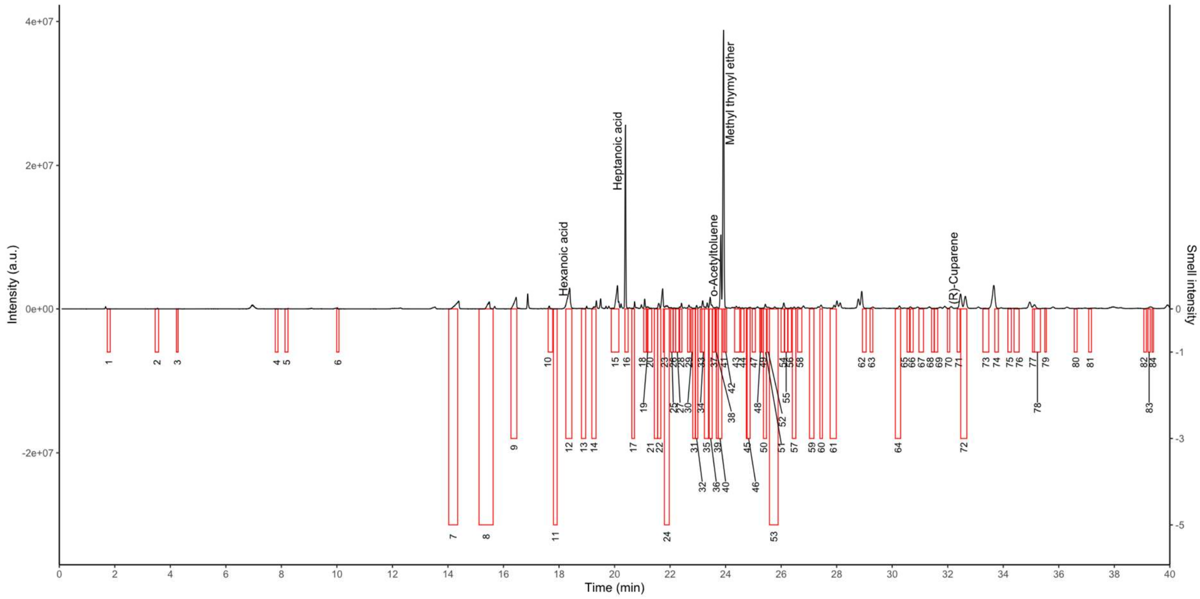

Supplementary Figure 34. MS chromatogram (black) and olfactogram (red) for the sample M9 (subsample 3). The numeric labels refer to SI3.

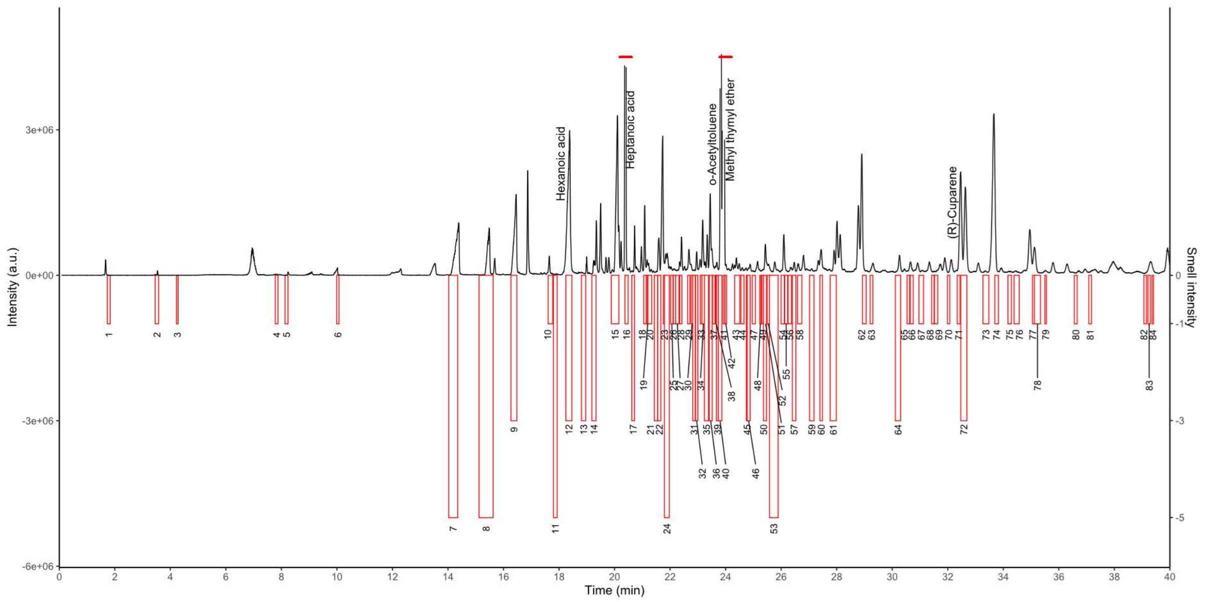

Supplementary Figure 35. MS chromatogram (black) and olfactogram (red) for the sample M9 (subsample 3) with a focus on the lower-intensity signals in the MS chromatogram. The numeric labels refer to SI3.

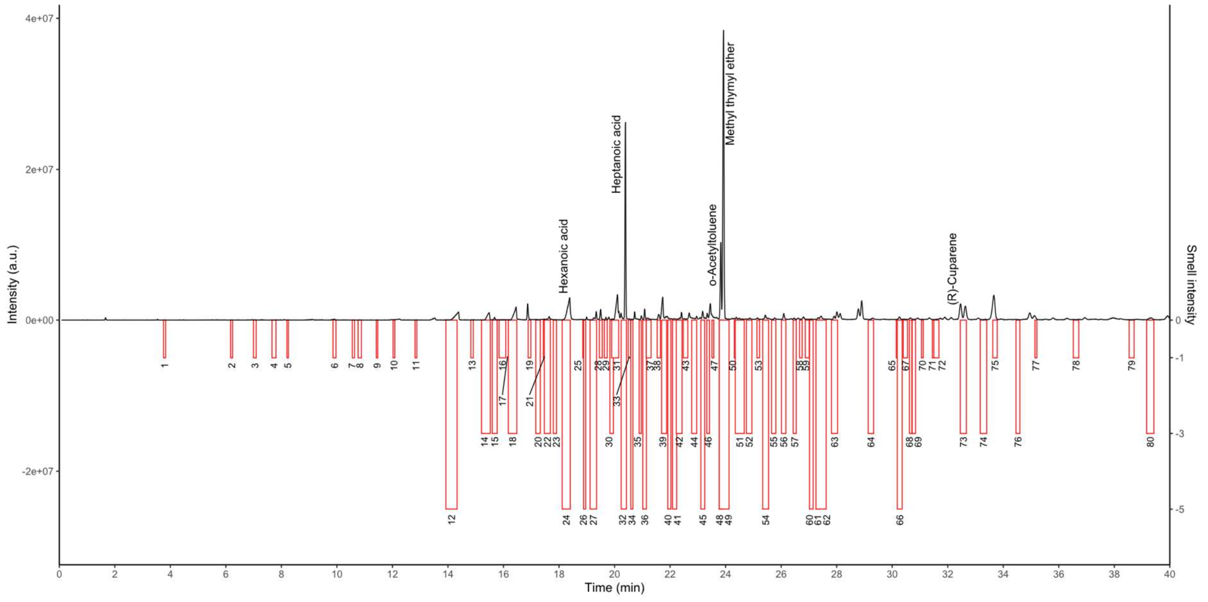

Supplementary Figure 36. MS chromatogram (black) and olfactogram (red) for the sample M9 (subsample 4). The numeric labels refer to SI3.

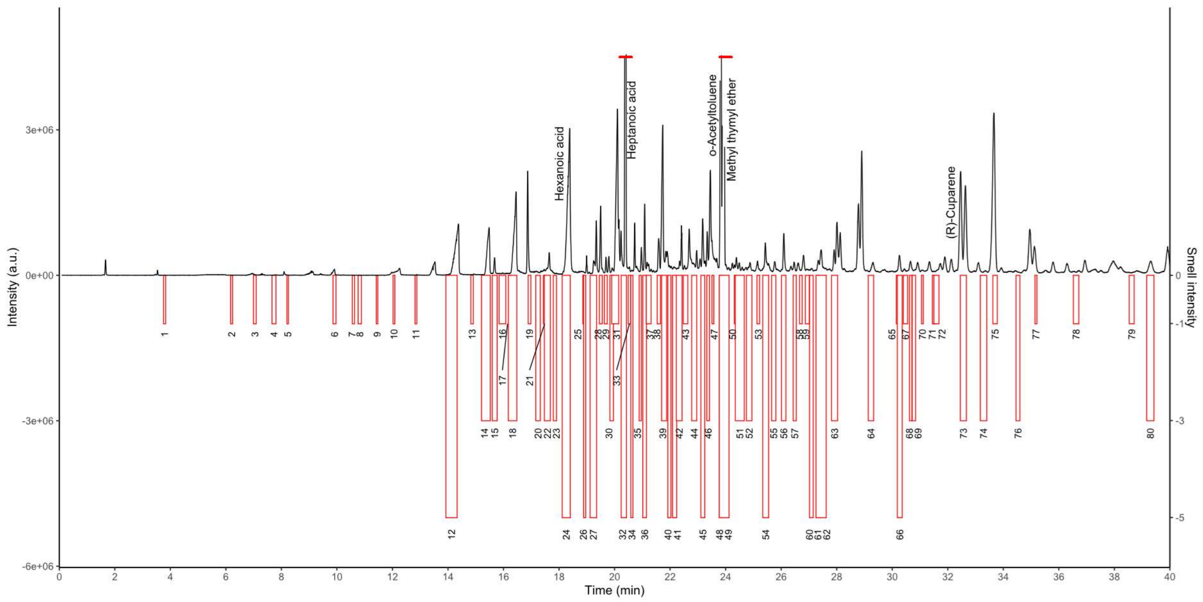

Supplementary Figure 37. MS chromatogram (black) and olfactogram (red) for the sample M9 (subsample 4) with a focus on the lower-intensity signals in the MS chromatogram. The numeric labels refer to SI3.

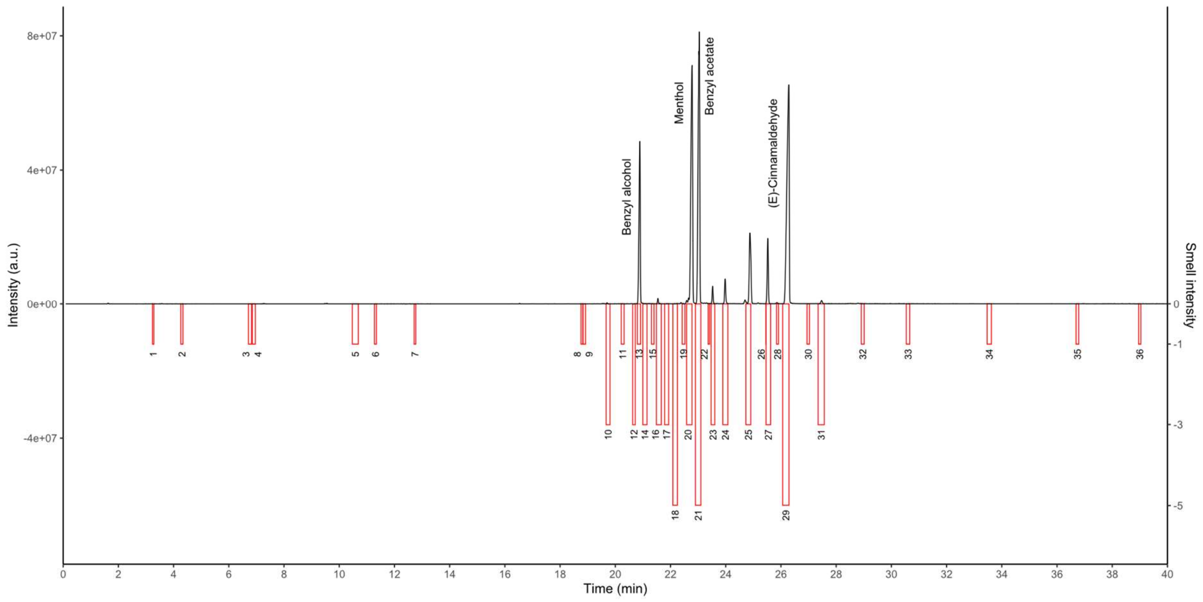

Supplementary Figure 38. MS chromatogram (black) and olfactogram (red) for the headspace analysis of the pest oil. The numeric labels refer to SI3.

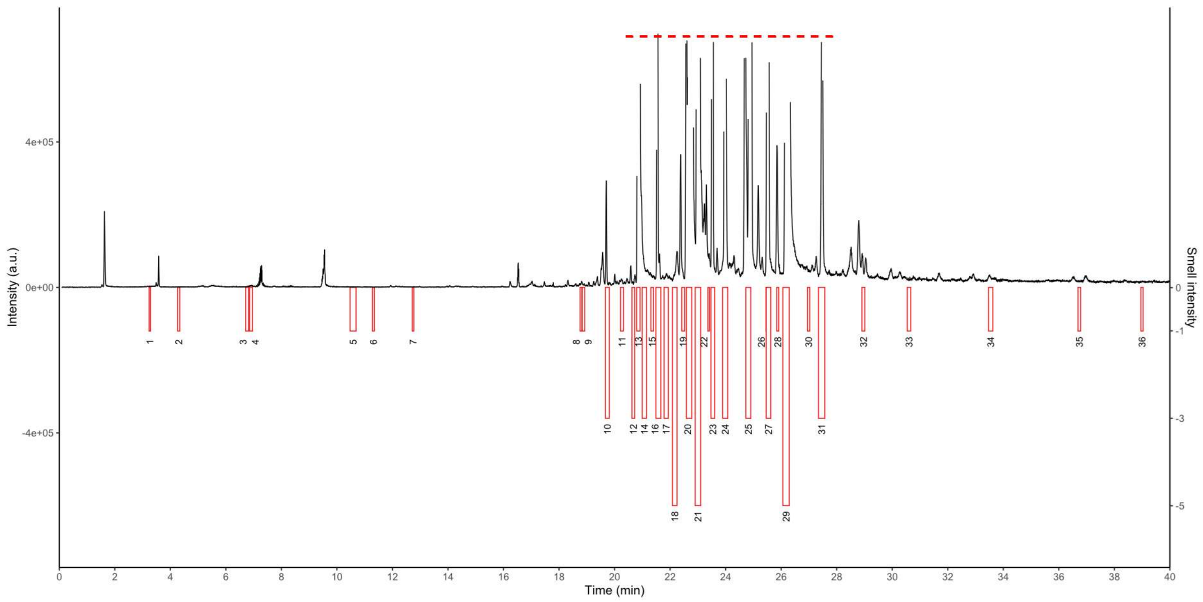

Supplementary Figure 37. MS chromatogram (black) and olfactogram (red) for the headspace analysis of the pest oil with a focus on the lower-intensity signals in the MS chromatogram. The numeric labels refer to SI3.

7. Radar plot for the pest oil odor profile

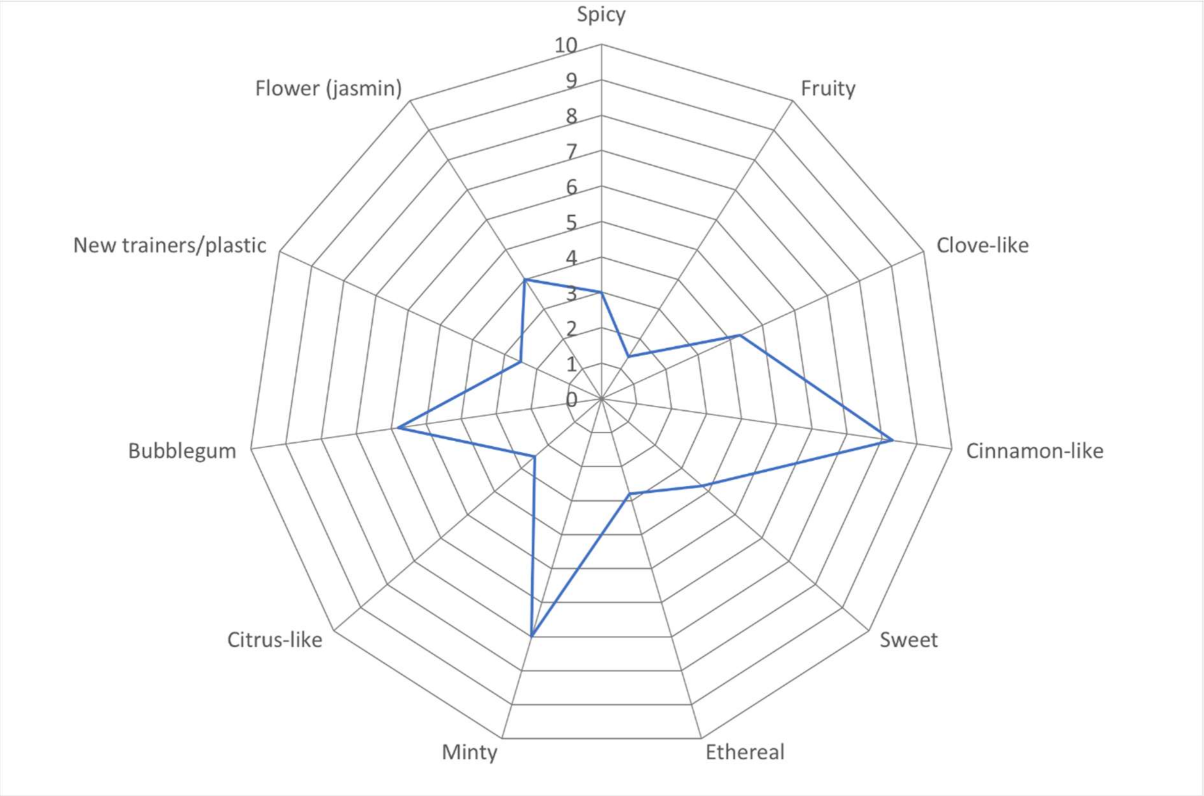

Supplementary Figure 38. Radar plot representing the odor profile of the pest oil. The labels correspond to odor quality descriptors. Perceived odor intensity is reported on a scale of 0-10,[19] where 0 indicates 'no odor' and 10 'very strong odor'.

8. References

1. Sandison, A. T. The use of natron in mummification in ancient Egypt. J. Near East. Stud. 22, 259-267 (1963).
2. Ikram, S. & Dodson, A. The Mummy in Ancient Egypt. (Thames & Hudson, 1998).
3. Serpico, M. & White, R. Oil, fat, and wax. in Ancient Egyptian Materials and methods (eds. Paul, N. & Shaw, I.) pp. 390–429 (Cambridge University Press, 2000).
4. David, A. R. Mummification. in Ancient Egyptian Materials and methods (ed: Paul T (eds. Nicholson, P. T. & Shaw, I.) pp. 372–389 (Cambridge University Press, 2000).
5. Abdel-Maksoud, G. & El-Amin, A.-R. A review on the materials used during the mummification processes in ancient Egypt. Mediterr. Archaeol. Archaeom. 11, 129–150 (2011).
6. Environmental Protection Agency: 1,4-Dichlorobenzene (para- Dichlorobenzene) Fact Sheet, https://www.epa.gov/sites/default/files/2016-09/documents/1-4-dichlorobenzene.pdf (Accessed 23rd July 2024).
7. Chourpiliadis, C. & Bhardwaj, A. Physiology, Respiratory Rate. (StatPearls, 2022).
8. Hallett, S., Toro, F. & Ashurst, J. Physiology, Tidal Volume. (StatPearls, 2023).
9. Health and Safety Executive. EH4O/2005 Workplace exposure limits. 1–74 (2011).
10. Skoog, D. A., Holler, J. F. & Crouch, S. R. Principles of Instrumental Analysis. (Cengage Learning, 2007).
11. Substance Evaluation Conclusion document for 1,2-dichlorobenzene. EC No 202-425-9. 1–51 (2020).

12. ECHA. Chlorpyrifos Draft Risk Profile (2022) https://echa.europa.eu/documents/10162/8a51d7d9-e9a4-2513-e975-492fb70f825c (Accessed 23rd July 2024).

13. Jaubert, J.-N., Tapiero, C. & Dore, J. C. The field of odors: toward a universal language for odor relationships. Perfum. Flavorist 20, 1 (1995).

14. Huber, B. et al. Biomolecular characterization of 3500-year-old ancient Egyptian mummification balms from the Valley of the Kings. Sci. Rep. 13, 1–14 (2023).

15. Rageot, M. et al. Biomolecular analyses enable new insights into ancient Egyptian embalming. Nature 614, 287-293 (2023).

16. Dutoit, C. E., Binet, L., Fujii, H., Lattuati-Derieux, A. & Gourier, D. Nondestructive analysis of mummification balms in ancient Egypt based on EPR of vanadyl and organic radical markers of bitumen. Anal. Chem. 92, 15445–15453 (2020).

17. Clark, K. A., Ikram, S. & Evershed, R. P. The significance of petroleum bitumen in ancient Egyptian mummies. Philos. Trans. R. Soc. A Math. Phys. Eng. Sci. 374, 20160229 (2016).

18. Conti, P. P. et al. Metal-organic frameworks for the capture of α-pinene traces. Chem. Commun. 59, 7064–7067 (2023)

19. Gilbert, A. What the Nose Knows: The Science of Scent in Everyday Life. (Crown Publishers, 2008).

20. Łucejko, J., Connan, J., Orsini, S., Ribechini, E. & Modugno, F. Chemical analyses of Egyptian mummification balms and organic residues from storage jars dated from the Old Kingdom to the Copto-Byzantine period. J. Archaeol. Sci. 85, 1–12 (2017).

21. Luebke, W. The good scents company. Chem Senses (1980).

22. http://www.flavornet.org (Accessed: 23rd July 2024).

23. Kreissl, J., Mall, V., Steinhaus, P. & M, S. Leibniz-LSB@TUM Odorant Database. (Leibniz Institute for Food Systems Biology, Technical University of Munich, Freising, 2002). https://www.leibniz-lsb.de/en/databases/leibniz-lsbtum-odorant-database/start.

24. Ahnfelt, N. O., Fors, H. & Wendin, K. Historical Continuity or Different Sensory Worlds? What we Can Learn about the Sensory Characteristics of Early Modern Pharmaceuticals by Taking Them to a Trained Sensory Panel. Ber. Wiss. 43, 412–429 (2020).

25. Tullett, W. *Smell and the Past: Noses, Archives, Narratives*, 1-12, (Bloomsbury Academic, 2023).

26. https://www.smellspedia.com/ (2024). (Accessed: 23rd July 2024).

27. Awad, A. H. A. et al. Indoor air fungal pollution of a historical museum, Egypt: a case study. Aerobiologia (Bologna). 36, 197–209 (2020).

28. Gutarowska, B., Skora, J., Zduniak, K. & Rembisz, D. Analysis of the sensitivity of microorganisms contaminating museums and archives to silver nanoparticles. Int. Biodeterior. Biodegrad. 68, 7–17 (2012).

29. Guiamet, P., Borrego, S. & Lavin, P. Biofouling and biodeterioration in materials stored at the Historical Archive of the Museum of La Plata, Argentine and at the National Archive of the Republic of Cuba. Colloids Surfaces B Biointerfaces 85, 229-234 (2011).

30. Saleem, A. & El-Said, A. Proteolytic activity of beef luncheon fungi as affected by incorporation of some food preservatives. Acta Microbiol. Immunol. Hung. 56, 417–426 (2009).

31. Ismael, S., Omar, A. & Maher, M. Comparative Inhibition Study by Nanomaterial, Plant Extract and Chemical Microcide on the Screaming Mummy in Egyptian Museum Store. Heritage 4, 2481-2493 (2021).
