# Supplementary material for: Ancient Egyptian Mummified Bodies: Cross-Disciplinary Analysis of Their Smell
Source: J Am Chem Soc. 2025 Feb 13;147(8):6633–43. doi: 10.1021/jacs.4c15769 (PMC11869298; doi:10.1021/jacs.4c15769)
Supplement: Supplementary file 5 — ja4c15769_si_005.pdf [file ja4c15769_si_005.pdf]

Ancient Egyptian Mummified Bodies: Cross-disciplinary Analysis of Their Smell

Emma Paolin[a], Cecilia Bembibre[b], Fabiana Di Gianvincenzo[a], Julio Cesar Torres-Elguera[c], Randa Deraz[a], Ida Kraševec[a], Ahmed Abdellah[d], Asmaa Ahmed[d], Irena Kralj Cigić[a], Abdelrazek Elnaggar[a,e], Ali Abdelhalim[d,e], Tomasz Sawoszczuk[c], Matija Strlič[a,b]*

[a] Heritage Science Laboratory Ljubljana, Faculty of Chemistry and Chemical Technology, University of Ljubljana, Večna pot 113, Ljubljana 1000, Slovenia

[b] Institute for Sustainable Heritage, University College London, 14 Upper Woburn Place, London WC1H 0NN, U.K.

[c] Department of Microbiology, Institute of Quality Sciences and Product Management, Krakow University of Economics, Henryka Sienkiewicza 4, Krakow 30 - 033, Poland

[d] The Egyptian Museum in Cairo, Cairo 4272083, Egypt

[e] Faculty of Archaeology, Ain Shams University, Abbasiya Cairo 11566, Egypt

Supplementary Information 5

Informed Consent for Participation in Sensory Analysis

Informed consent for participation in SENSORY ANALYSIS

Research: **ODOTHEKA project**

1. You are kindly invited to take part in the research project ODOTHEKA. The research project is carried out by the Faculty of Chemistry and Chemical Technology of the University of Ljubljana under the leadership of Prof Dr Matija Strlič and at the Department of Microbiology of the University of Economics in Krakow under the leadership of Prof Dr Tomasz Sawoszczuk. The purpose of the research is to understand and preserve the smell of historical objects and to develop an international archive of heritage smells. This will be performed by chemical and sensor analysis of the volatile compounds emitted by an object, and evaluation of its significance.

2. If you decide to participate in the research, your task will be to contribute to the sensory analysis at the Egyptian Museum in Cairo where you will be asked to smell the odour of 10 mummies.

3. The duration of sensory analysis will be approximately four hours.

4. Participation in the research consists of smelling the odour emitted by 10 ancient mummified bodies. Preliminary chemical analysis with a SPME sampling fibre on 8 out of 10 mummies, showed that some potentially harmful compounds are present in the object headspace, such as chlorinated compounds 1,2-dichlorobenzene, 1,4-dichlorobenzene (used as fumigants against moths and mould[1]) and a broad-spectrum organophosphate pesticide Chlorpyrifos.
   At a normal respiratory rate of 12-20 breaths per minute[2] and 400-500 mL of air per breath[3], in an environment with a substance concentration at the short-term exposure limit (STEL)[4], the maximum permitted inhaled dose is 46 mg for 1,2-dichlorobenzene, 9 mg for 1,4-dichlorobenzene and 0.09 mg for Chlorpyrifos (calculated as a product of STEL, exposure time of 15 min, respiratory rate and breath volume). These concentrations are 4-7 orders of magnitude bigger than the ones detected in the preliminary analysis, the activity thus representing a very low risk to the participant.

Substance	Health effects	Short-term exposure limit (15-minute reference period)[3]	Max permitted inhaled dose in 15 min
1,2-Dichlorobenzene	Eye and respiratory irritation at high concentrations, no concern for the carcinogenic potential[5]	50 ppm or 306 mg/m^3	46 mg
1,4-Dichlorobenzene	Eye and respiratory irritation at high concentrations6, harmful effects on the liver, skin, and central nervous system at long-term exposure[1]	10 ppm or 60 mg/m^3	9 mg
Chlorpyrifos	Nausea, dizziness, confusion, respiratory paralysis at high concentrations, developmental neurotoxicity[7]	0.6 mg/m^3	0.09 mg

5. Your participation in the research is completely voluntary and you may choose to terminate it without consequences at any time.

6. We will do all in our power to protect your privacy. The record of your experience and the accompanying demographic information (age and gender) will be stored under a research code. Only the aggregate results will be made publicly available. Your identity will never be disclosed.

7. Should you have any questions, please contact Emma Paolin, Emma.Paolin@fkkt.uni-lj.si, Faculty of Chemistry and Chemical Technology, University of Ljubljana.

By signing this statement, I guarantee that I have read the statement and that I have been given an opportunity to pose questions in relation to the research. I hereby grant consent to my participation in the research ODOTHEKA and agree to the use of results for the purpose of teaching and scientific research. I have read and understood the above information and I am aware of the potential health risk.

__________________ _____________________________ ______________

Name and surname Signature of the participant Date

__________________ _____________________________ ______________

Name and surname Signature of the administrator Date

__________________ _____________________________ ______________

Name and surname Signature of the person conducting the research Date

References:

1. Environmental Protection Agency. *1,4-Dichlorobenzene (para-Dichlorobenzene) Fact Sheet*, https://www.epa.gov/sites/default/files/2016-09/documents/1-4-dichlorobenzene.pdf (Accessed 23rd July 2024).
2. Chourpiliadis, C. & Bhardwaj, A. *Physiology, Respiratory Rate*. (StatPearls, 2022).
3. Hallett, S., Toro, F. & Ashurst, J. *Physiology, Tidal Volume.* (StatPearls, 2023).
4. Health and Safety Executive. EH40/2005 Workplace exposure limits. 1–74 (2011).
5. National Public Health Center, *Substance Evaluation Conclusion as required by REACH Article 48 and Evaluation Report for 1,2-dichlorobenzene.* (2020) https://echa.europa.eu/documents/10162/6359460c-62a2-010f-e361-d87e38ec6c25 (Accessed 23rd July 2024).
6. European Communities, *1,2-Dichlorobenzene, Summary Risk Assessment Report*, Special Publication I.04.218. (2004). https://echa.europa.eu/documents/10162/a2738f4a-70cf-4c33-bd6d-39d1523ca0e4 (Accessed 23rd July 2024).
7. ECHA*, Draft proposal for listing chlorpyrifos in Annex A to the Stockholm Convention on Persistent Organic Pollutants*. (2020). https://echa.europa.eu/documents/10162/bb4342c2-64f1-d184-e8d8-334f02842f9b (Accessed 23rd July 2024).
